# Human amniotic membrane inhibits migration and invasion of muscle-invasive bladder cancer urothelial cells by downregulating the FAK/PI3K/Akt/mTOR signalling pathway

**DOI:** 10.1038/s41598-023-46091-2

**Published:** 2023-11-06

**Authors:** Aleksandar Janev, Taja Železnik Ramuta, Urška Dragin Jerman, Hristina Obradović, Urška Kamenšek, Maja Čemažar, Mateja Erdani Kreft

**Affiliations:** 1 Institute of Cell Biology, Faculty of Medicine, University of Ljubljana, Ljubljana, Slovenia; 2 Department of Experimental Oncology, Institute of Oncology Ljubljana, Ljubljana, Slovenia

**Keywords:** Cancer, Cell biology, Urology

## Abstract

Bladder cancer is the 10th most commonly diagnosed cancer with the highest lifetime treatment costs. The human amniotic membrane (hAM) is the innermost foetal membrane that possesses a wide range of biological properties, including anti-inflammatory, antimicrobial and anticancer properties. Despite the growing number of studies, the mechanisms associated with the anticancer effects of human amniotic membrane (hAM) are poorly understood. Here, we reported that hAM preparations (homogenate and extract) inhibited the expression of the epithelial–mesenchymal transition markers N-cadherin and MMP-2 in bladder cancer urothelial cells in a dose-dependent manner, while increasing the secretion of TIMP-2. Moreover, hAM homogenate exerted its antimigratory effect by downregulating the expression of FAK and proteins involved in actin cytoskeleton reorganisation, such as cortactin and small RhoGTPases. In muscle-invasive cancer urothelial cells, hAM homogenate downregulated the PI3K/Akt/mTOR signalling pathway, the key cascade involved in promoting bladder cancer. By using normal, non-invasive papilloma and muscle-invasive cancer urothelial models, new perspectives on the anticancer effects of hAM have emerged. The results identify new sites for therapeutic intervention and are prompt encouragement for ongoing anticancer drug development studies.

## Introduction

According to the latest Global Cancer Statistics database, bladder cancer is the 10th most commonly diagnosed cancer worldwide^1^. At the time of diagnosis, 25% of patients have muscle-invasive bladder cancer (MIBC), while 75% of patients have non-muscle-invasive bladder cancer (NMIBC)^2,3^. Despite the favourable prognosis, high recurrence rates (up to 70%) and progression to MIBC (10–30%) are still observed in patients with NMIBC^3–5^. On the other hand, patients with MIBC have poorer prognosis as they often develop metastases^6^. Despite major advances in the treatment of some other cancers in recent years, there has not yet been a major breakthrough in standard treatment options for bladder cancer. Therefore, there is an urgent need for developing new, more effective and targeted therapeutic approaches.

Focal adhesion kinase (FAK) is a non-receptor cytoplasmic tyrosine kinase localised at focal adhesion sites. In addition to its kinase function, FAK also functions as a scaffold protein and is involved in various protein–protein interactions^7^. Cell surface receptors, such as integrins, G-protein-coupled receptors, cytokine receptors and growth factor receptors, relay on extracellular signals to the FAK protein, which in turn, triggers various signalling pathways important for cell adhesion, motility, growth, proliferation and survival^7–9^. Many of these processes are dysfunctional in cancer. It is often observed that upregulation of FAK signalling is associated with poorer prognosis and tumour progression, even in bladder cancer^9,10^.

The human amniotic membrane (hAM) is an extraembryonic membrane that surrounds the embryo. It consists of human amniotic epithelial cells (hAEC), thick basal lamina and non-vascularised connective tissue with human amniotic mesenchymal stromal cells (hAMSC)^11–13^. In addition to extracellular macromolecules, such as collagens, laminins, proteoglycans and hyaluronic acid, hAM is a source of stem cells and various active signalling molecules, including interleukin 6 (IL-6), tumour necrosis factor alpha (TNF-α), transforming growth factor beta (TGF-β), epidermal growth factor (EGF), interferon gamma (IFN-γ) and tissue inhibitor of metalloproteinases 1–4 (TIMP1-4)^13–15^. In recent years, a growing body of research has shown that the hAM has anticancer potential^16–21^. Namely, it has been shown that hAM preparations, hAEC and hAMSC as well as hAM scaffolds, inhibit the proliferation of muscle-invasive bladder cancer urothelial cells. In addition to the effect on proliferation, hAM scaffolds also reduce the invasive potential and the expression of mesenchymal markers (Snail, Slug, N-cadherin) in cancer urothelial cells^18^. Furthermore, we were the first to demonstrate that the hAM homogenate decreased the adhesion of cancer urothelial cells, impeded their growth dynamics and disrupted the 3D structure of bladder cancer spheroids, while it had no such deleterious effects on normal urothelial cells^22^.

Despite the growing number of in vitro and in vivo studies, the cell biological and molecular mechanisms underlying the anticancer effects of hAM are poorly understood. To the best of our knowledge, the effects of hAM homogenate and extract on signalling pathways involved in cell adhesion, migration and invasion of bladder cancer urothelial cells have not yet been investigated. Therefore, the aim of our study was to investigate the anticancer mechanism of action of hAM preparations in various in vitro models of bladder cancer and normal urothelium.

## Results

### hAM preparations hinder the migration of bladder cancer urothelial cells in a time- and dose-dependent manner

**Figure 1:**
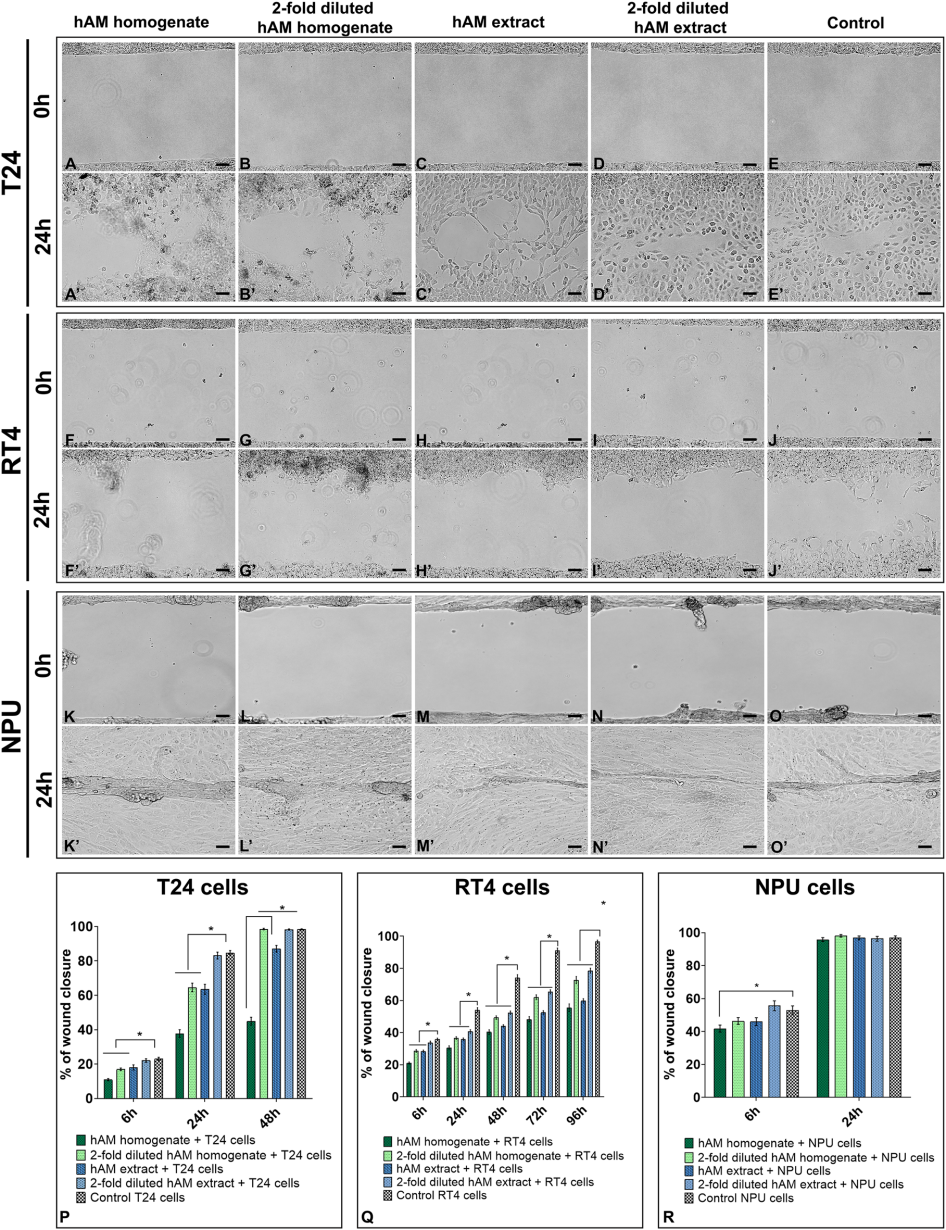
Dose- and time-dependent inhibition of bladder cancer cell migration by hAM preparations. (**A**–**E′**) Representative images of wound healing of T24 cells. (**F**–**J′**) Representative images of wound healing of RT4 cells. (**K**–**O**′) Representative images of wound healing of NPU cells. (P-R) Percentage of wound healing of T24, RT4 and NPU cells, respectively. Data are presented as mean ± standard error of the mean (SEM) of at least three independent experiments. *P < 0.05 vs. control group. Scale bars 50 µm.

To investigate whether hAM preparations affect the migration rate, cancer and normal urothelial cells were treated with different concentrations of hAM homogenate and hAM extract or an appropriate culture medium (controls) (Fig. 1A–R). Our results showed that hAM homogenate and extract significantly decreased the migration of muscle-invasive bladder cancer urothelial T24 cells in a time-dependent manner (Fig. 1A–A′,C–C′,P). Although to a lesser extent, we showed that even twofold diluted hAM homogenate significantly inhibited the migration of T24 cells after 6- and 24-h treatment (Fig. 1B–B′,D–D′,P). In the same way, we showed that hAM preparations inhibited the migration of non-invasive urothelial papilloma RT4 cells in a dose- and time-dependent manner (Fig. 1F–J′,Q). However, a significant decrease in the migration rate of normal urothelial cells was only observed after 6 h of treatment with hAM homogenate (Fig. 1R). This inhibitory effect disappeared after 24 h of incubation, suggesting that hAM homogenate does not permanently hinder the migration of normal urothelial NPU cells (Fig. 1K–O′,R).

**Figure 2:**
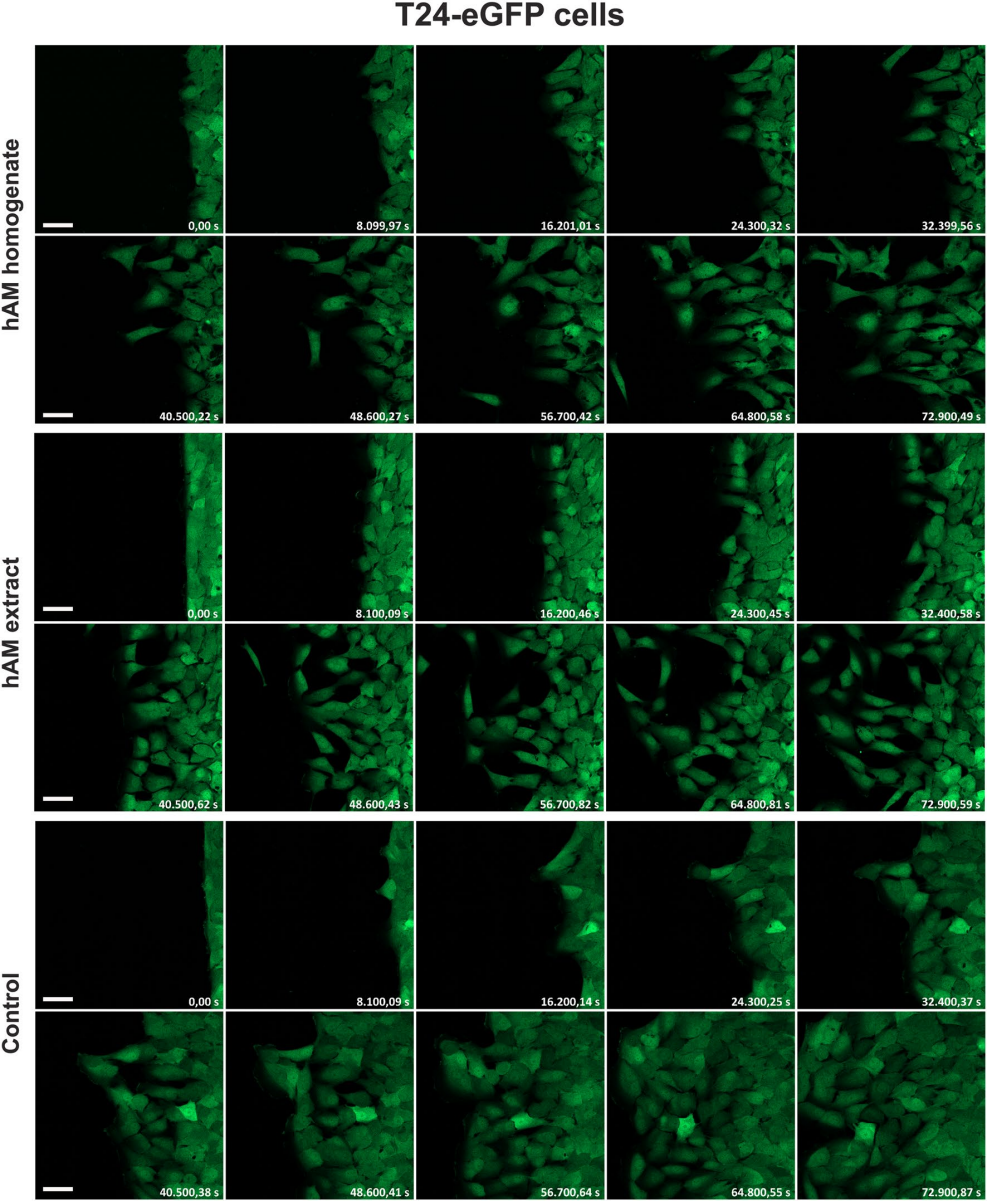
hAM homogenate and extract induce a shift from collective to individual cell migration, thereby altering the migration pattern of muscle-invasive cancer urothelial cells. T24-eGFP cells treated with hAM homogenate and extract migrate individually, whereas T24-eGFP cells treated with appropriate culture medium without hAM homogenate migrate collectively. The presented images were acquired consecutively every 2 h and 15 min (shown in seconds) after the treatment. Shown is one representative experiment out of three independent experiments with different hAM preparations. Scale bars 50 µm.

**Figure 3:**
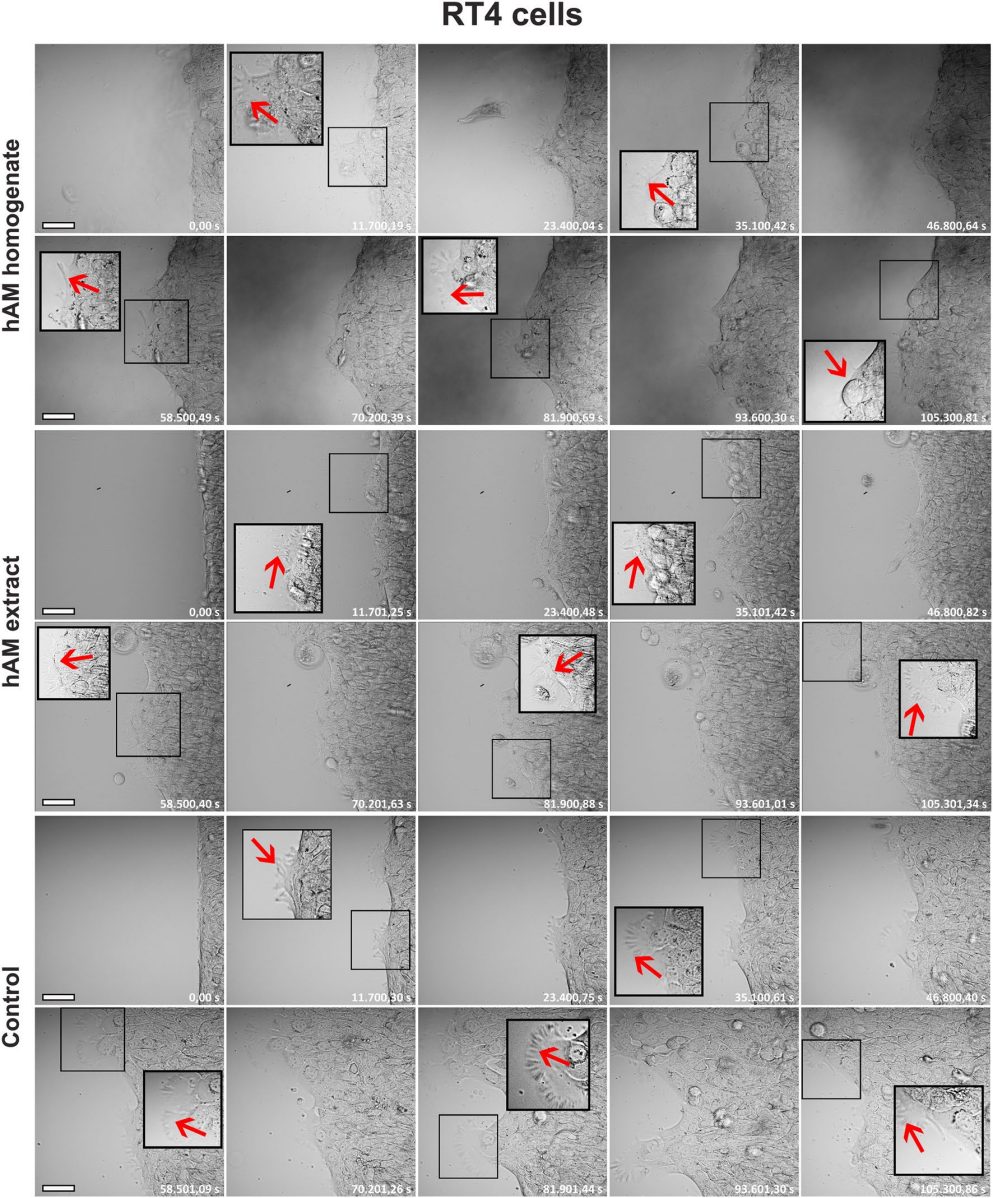
hAM homogenate and extract suppress the migration rate of non-invasive urothelial papilloma RT4 cells by decreasing the amount of lamellipodia and filopodia along the leading edge. RT4 cells treated with hAM homogenate, hAM extract or an appropriate culture medium (control) migrate collectively and maintain an epithelial morphology. However, migrating RT4 cells, treated with hAM preparations, contain less lamellipodia and filopodia (red arrows) along the leading edge than untreated control RT4 cells. Larger framed inset with black lines are the enlarged images of the corresponding smaller framed inserts. The presented images were acquired consecutively every 3 h and 15 min (shown in seconds) after the treatment. Shown is one representative experiment out of three independent experiments with different hAM preparations. Scale bars 50 µm.

**Figure 4:**
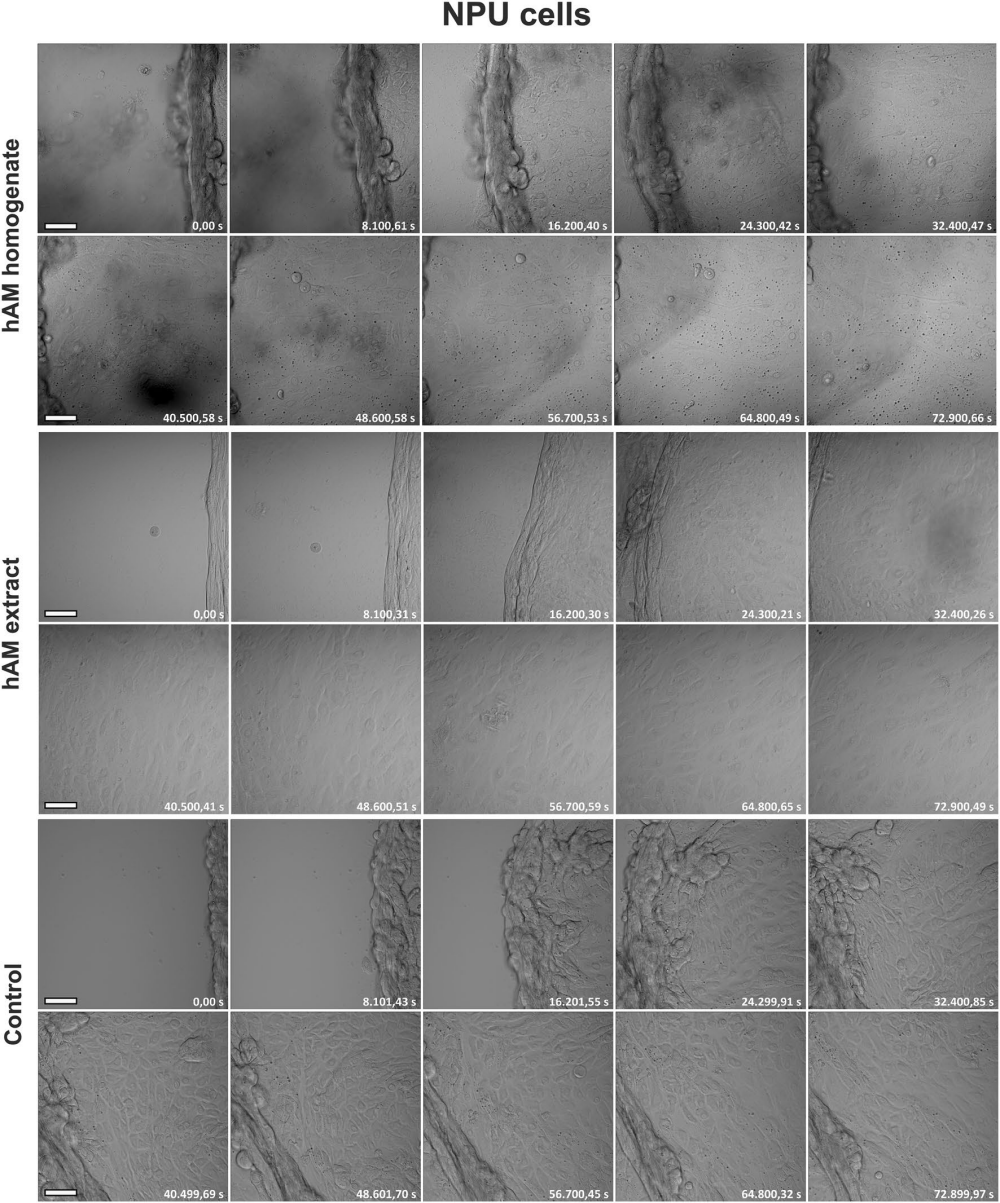
hAM preparations do not affect the morphology and the pattern of migration of normal urothelial cells. NPU cells treated with hAM homogenate, hAM extract or an appropriate culture medium (control) migrate collectively and maintain an epithelial morphology. The presented images were acquired consecutively every 2 h and 15 min (shown in seconds) after the treatment. Shown is one representative experiment out of three independent experiments with different hAM preparations. Scale bars 50 µm.

Next, we investigated whether hAM homogenate and extract affect the morphology of migrating bladder cancer and normal urothelial cells. For this purpose, we used continuous time-lapse live imaging of T24-eGFP, RT4 and NPU cells treated with hAM homogenate or extract. Our results showed that the T24-eGFP cells treated with hAM homogenate and extract migrated individually (Fig. 2 and Supplementary Movie S1). They had an elongated shape with many intercellular spaces between them. At the leading edge, we observed a loss of directional persistence and random cell migration, especially in the T24-eGFP cells treated with hAM homogenate (Fig. 2 and Supplementary Movie S1). On the other hand, T24-eGFP cells treated with culture medium without hAM preparations migrated collectively as a sheet of cells. We did not observe intercellular spaces between the migrating cells, as the intercellular connections were maintained throughout the migration period (Fig. 2 and Supplementary Movie S2). RT4 cells treated with hAM homogenate, hAM extract or culture medium maintained their collective epithelial migration (Fig. 3 and Supplementary Movie S2). However, we observed a lesser amount of lamellipodia and filopodia along the leading edge of RT4 cells treated with hAM homogenate and hAM extract compared to control RT4 cells. This observation suggests that hAM homogenate and hAM extract suppress the migration rate of RT4 cells by regulating lamellipodia and filopodia formation at the leading edge (Fig. 3 and Supplementary Movie S2). On the other hand, we showed that hAM homogenate and hAM extract have no effect on the morphology and migration pattern of NPU cells (Fig. 4 and Supplementary Movie S3).

### hAM preparations decrease the invasion rate of muscle-invasive bladder cancer urothelial cells in a dose-dependent manner

**Figure 5:**
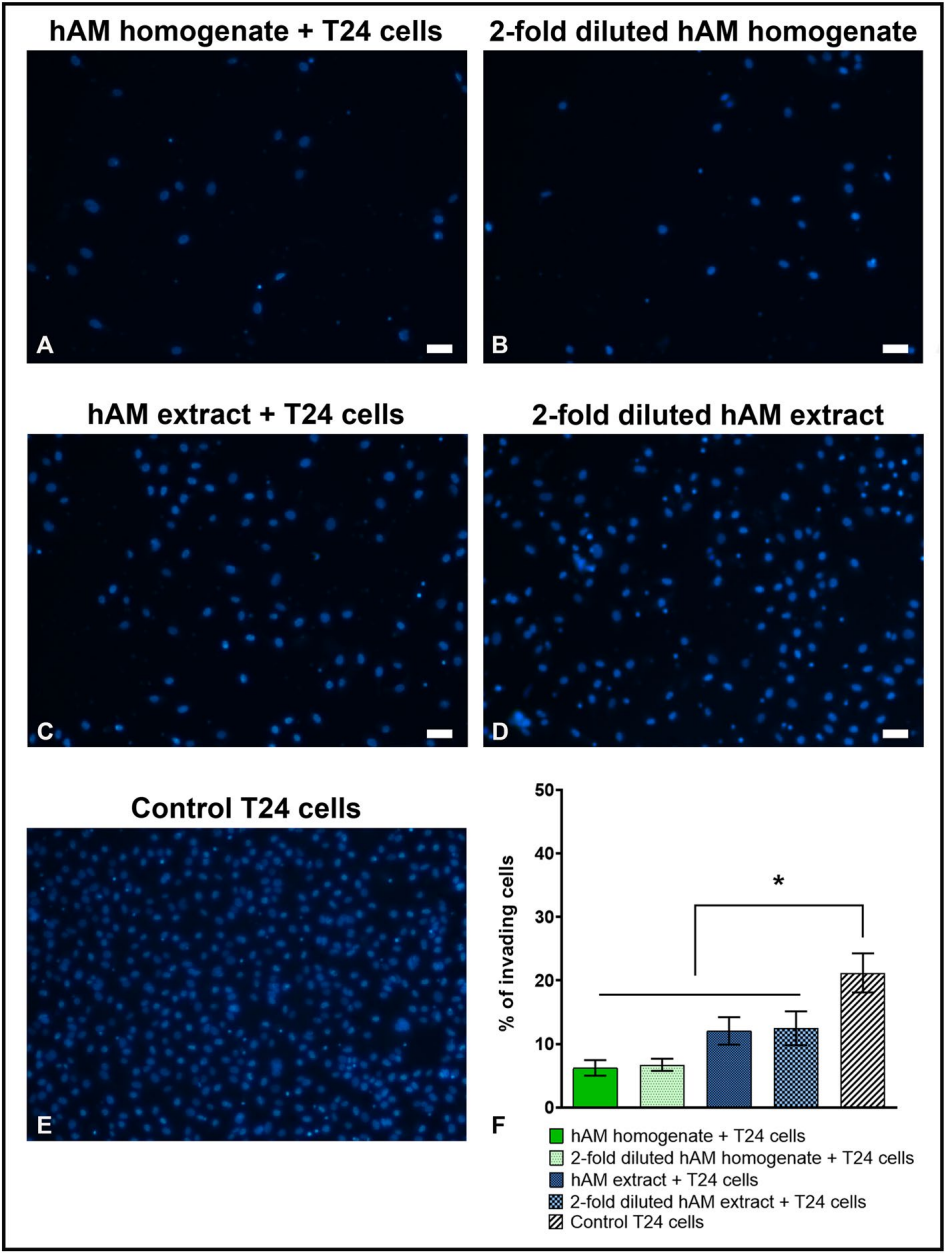
hAM preparations reduce the invasive capacity of T24 cells in a dose-dependent manner. (**A**–**E**) T24 cells treated with (**A**) hAM homogenate, (**B**) twofold diluted hAM homogenate, (**C**) hAM extract and (**D**) twofold diluted hAM extract is less invasive than (**E**) control T24 cells treated with culture medium. (**F**) Percentage of invading T24 cells in the lower transwell chamber after 24-h treatment with hAM preparations or culture medium. Data are presented as mean ± SEM of at least three independent experiments. *P ≤ 0.05 vs. control group. Scale bars 50 µm.

Next, we investigated the effect of hAM homogenate and hAM extract on the invasion rate of muscle-invasive bladder cancer urothelial cells T24. Our results showed that hAM preparations inhibited the invasion of T24 cells in a dose-dependent manner. Namely, cells treated with hAM homogenate and hAM extract showed a lower invasion capacity than control T24 cells treated with culture medium without hAM preparations (Fig. 5A,C,E,F). We were also able to show that twofold diluted hAM homogenate and hAM extract also decreased the invasion of T24 cells (Fig. 5B,D,F).

### hAM preparations inhibit FAK expression at gene and protein level in bladder cancer urothelial cells

**Figure 6:**
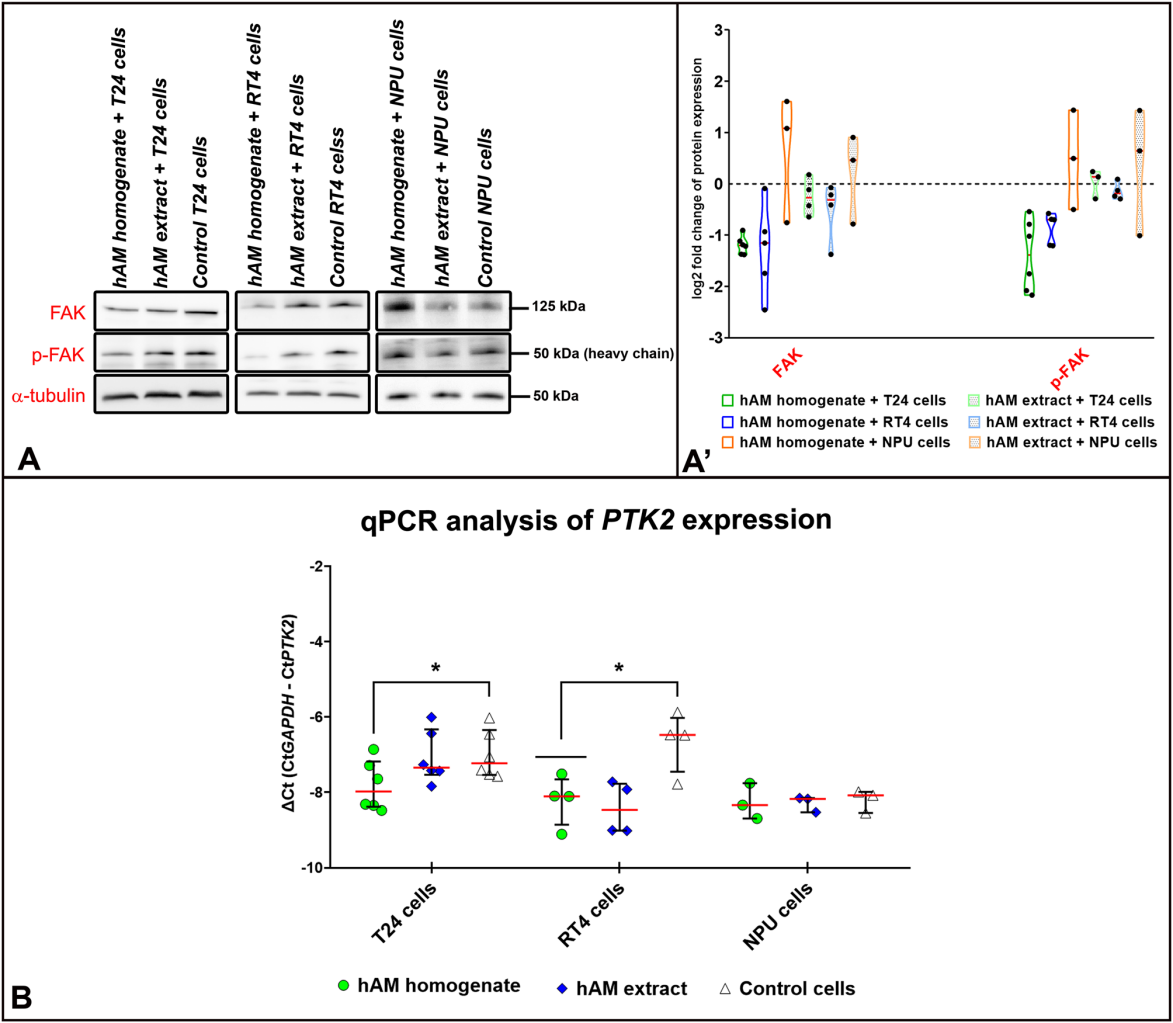
hAM homogenate and extract down-regulate FAK expression in bladder cancer urothelial cells but not in normal urothelial cells. (**A**) Western blot analysis of FAK and p-FAK in T24, RT4 and NPU cells treated for 24 h with hAM homogenate, hAM extract or appropriate culture medium (control samples) without hAM preparations. (**A′**) The expression and phosphorylation of FAK were normalised to the level of α-tubulin. In addition, the expression and phosphorylation of FAK in the treated samples were normalised to the control samples and these values are presented as log2 fold change (log2FC). The results are expressed as median (solid black line) with data range (minimum and maximum). The dashed black lines represent the normalised value of the control sample (0). The results were obtained on the basis of 3–6 biological replicates. The most representative blot of the mean effect is presented. The original blots are presented in Supplementary Fig. 2. The absence of full-length membranes is due to the cropping of the membranes prior to hybridization with primary antibodies. * p ≤ 0.05. (**B**) qPCR analysis of *PTK2* expression in T24, RT4 and NPU cells treated for 24 h with hAM homogenate, hAM extract or appropriate culture medium without hAM preparations (control samples). *PTK2* expression was normalised to *GAPDH* levels. The qPCR results were presented as ΔCt (Ct*GAPDH* − Ct*PTK2*). Each dot in the graph represents the value of one biological sample. The solid red lines show the median and the solid black lines represent the interquartile range. *p ≤ 0.05.

Since FAK is one of the activators of several signalling pathways involved in several important cellular processes such as cell adhesion, migration, proliferation and invasion, we aimed to investigate the effect of hAM preparations on its expression and phosphorylation. For this purpose, T24, RT4 and NPU cells were treated with hAM homogenate, hAM extract or appropriate culture medium (controls) for 24 h. The expression of FAK and its phosphorylation status were assessed by Western blot analysis (Fig. 6A,A′), while total mRNA expression was assessed by qPCR (Fig. 6B).

The results of our study showed that hAM homogenate significantly downregulated the expression and phosphorylation of FAK in muscle-invasive bladder cancer urothelial cells T24 (Fig. 6A,A′). We also showed that hAM homogenate not only affected FAK expression at the protein level, but also downregulated *PTK2* gene expression (Fig. 6B). Moreover, hAM extract down-regulated FAK expression in T24 cells, albeit to a lesser extent than hAM homogenate (Fig. 6A–A′). However, hAM extract had no effect on phosphorylated FAK and the expression of *PTK2* in T24 cells (Fig. 6A,B).

Similar to muscle-invasive bladder T24 cells, hAM homogenate and hAM extract strongly inhibited FAK expression in non-invasive urothelial papilloma RT4 cells (Fig. 6A–A′). This inhibitory effect was even more pronounced on gene level, since both hAM preparations significantly downregulated the *PTK2* gene expression (Fig. 6B). Furthermore, hAM homogenate also significantly reduced the expression of phosphorylated FAK, whereas hAM extract had no significant effect (Fig. 6A–A′).

In normal urothelial NPU cells, we showed that hAM homogenate and hAM extract did not affect the expression of *PTK2* and FAK, and also did not affect FAK phosphorylation (Fig. 6A–B).

### hAM homogenate downregulates the PI3K/Akt/mTOR signalling pathway in muscle-invasive bladder cancer urothelial cells

**Figure 7:**
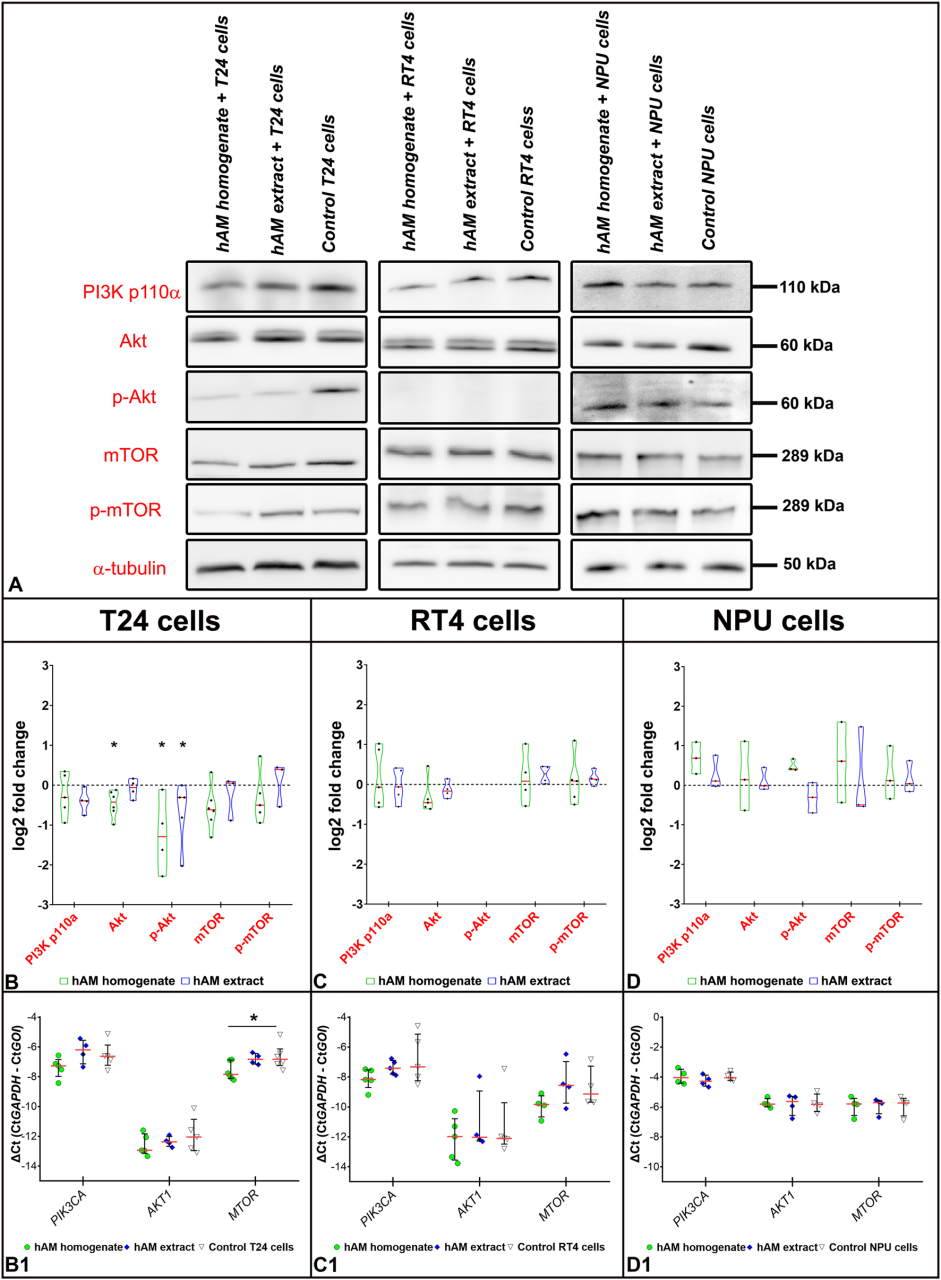
hAM homogenate inhibits the PI3K/Akt/mTOR pathway in muscle-invasive bladder cancer cells T24. (**A**–**D**) Western blot analysis of PI3K p110α, Akt, p-Akt, mTOR and p-mTOR expression in bladder cancer and normal urothelial cells after treatment with hAM homogenate, extract and appropriate culture medium (control samples). The expression and phosphorylation of the target proteins were normalised to the level of α-tubulin. In addition, the expression and phosphorylation of the target proteins in the treated samples were normalised to the control samples and the data presented as log2 fold change (log2FC). Data are presented as median (solid red line) with data range (minimum and maximum). The dashed black lines represent the normalised value of the control sample (0). Western blot analysis results were obtained on the basis of 3–6 biological replicates. The most representative blot of the mean effect is presented. The original blots are presented in Supplementary Fig. 2. The absence of full-length membranes is due to the cropping of the membranes prior to hybridization with primary antibodies. (**B1**–**D1**) qPCR analysis of the expression of *PIK3CA*, *AKT1* and *MTOR* in cancer and normal bladder cells treated for 24 h with hAM homogenate, hAM extract or appropriate culture medium without hAM preparations (control samples). The expression of the genes of interest (GOI) was normalised to the *GAPDH* levels. The qPCR results were presented as ΔCt (Ct*GAPDH* − CtGOI). Each point in on the graphs (**B1**–**D1**) represents the value of a biological sample. The solid red lines show the median and the solid black lines represent the interquartile range. *p ≤ 0.05.

Since FAK is one of the activators of the PI3K/Akt/mTOR signalling pathway, we were interested in knowing whether hAM preparations exert their effect by regulating the expression of genes and proteins involved in the PI3K/Akt/mTOR pathway. We have shown that hAM homogenate downregulates the protein levels of PI3K p110α, Akt and mTOR in T24 cells (Fig. 7A,B). Furthermore, we have demonstrated that the expression of the corresponding genes *PIK3CA*, *AKT1* and *MTOR* was reduced after treatment of T24 cells with hAM homogenate (Fig. 7B1). In addition to protein and gene expression, hAM homogenate also decreased Akt and mTOR phosphorylation levels in muscle-invasive bladder cancer T24 cells (Fig. 7A,B).

The effect of hAM homogenate and extract on the expression of genes and proteins involved in the PI3K/Akt/mTOR pathway was somewhat different in transitional cell papilloma RT4 cells. Although the hAM preparations inhibited the total amount of FAK, they had no significant effect on the expression of other genes and proteins that are part of the above-mentioned signalling pathway (Fig. 7A,C–C1). We did not detect any phosphorylated form of Akt in transitional cell papilloma RT4 cells, which is consistent with the findings of other research groups^23–25^.

Furthermore, we showed that the hAM preparations had the opposite effect on normal urothelial cells. We demonstrated that in comparison with bladder cancer urothelial cells, hAM homogenate slightly increased the protein levels of PI3K p110α, mTOR and the amount of phosphorylated Akt and mTOR expression (Fig. 7A,D). In contrast, we observed slight decrease in the levels of phosphorylated Akt and mTOR after the treatment with hAM extract, but nevertheless this difference was not statistically significant compared to control NPU cells treated with culture medium (Fig. 7A,D). Using the qPCR method, we demonstrated that the hAM homogenate and extract did not affect the expression of *PIK3CA*, *AKT1* and *MTOR* genes in normal urothelial cells (Fig. 7D1).

### hAM homogenate decreases the expression of proteins, which are involved in the actin cytoskeleton reorganization of bladder cancer urothelial cells

**Figure 8:**
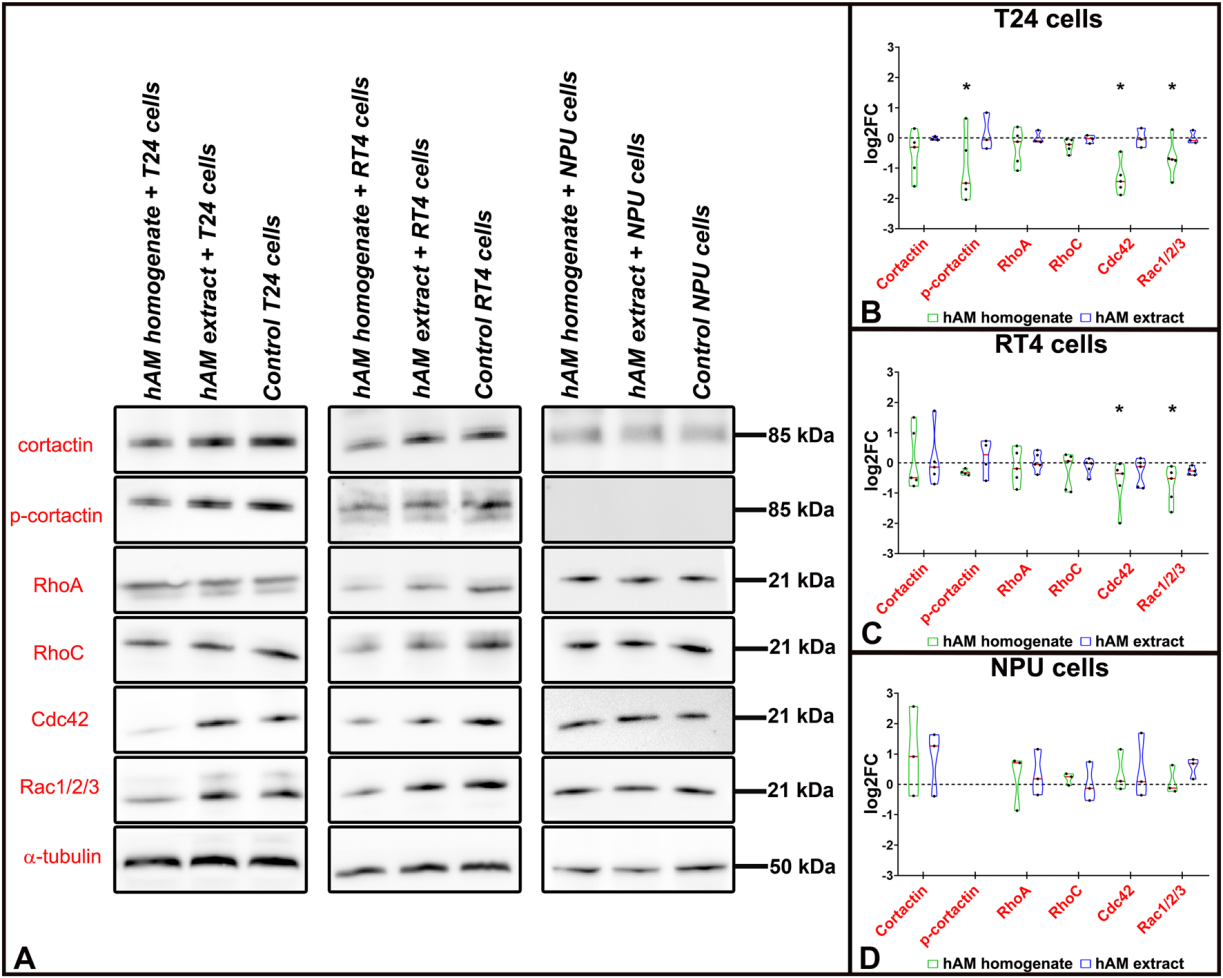
hAM homogenate inhibits the expression of proteins involved in actin cytoskeleton reorganisation. (**A**) Western blot analysis of cortactin, RhoA, RhoC, Cdc42, Rac1/2/3 and phosphorylation of cortactin in T24, RT4 and NPU cells treated for 24 h with hAM homogenate, hAM extract or appropriate culture medium (controls) without hAM preparations. (**B**–**D**) Quantification of relative protein expression and phosphorylation. The expression and phosphorylation of the target proteins were normalised to the α-tubulin values. In addition, the expression and phosphorylation of the target proteins in the treated samples were normalised to the control samples (culture medium without hAM preparation) and these values were presented as log2 fold change (log2FC). The results are as median (solid red line) with data range (minimum and maximum). The dashed black lines represent the normalised value of the control sample (0). Results were obtained on the basis of 3–6 biological replicates. The most representative blot of the mean effect is presented. The original blots are presented in Supplementary Fig. 2. The absence of full-length membranes is due to the cropping of the membranes prior to hybridization with primary antibodies. *p ≤ 0.05.

Since hAM preparations strongly reduced the migration of cancer urothelial cells, we further investigated their effect on the expression of key components required for efficient cell migration, such as cortactin, RhoA, RhoC, Cdc42 and Rac1. For this purpose, T24, RT4 and NPU cells were treated with hAM homogenate, hAM extract or appropriate culture medium (controls) for 24 h. The expression of proteins involved in cell migration were assessed by Western blot analysis (Fig. 8A–D).

We demonstrated that hAM homogenate significantly decreased the expression of Cdc42 and Rac1/2/3 in bladder cancer urothelial T24 and RT4 cells (Fig. 8A–C). Furthermore, we showed that hAM homogenate slightly reduced the cortactin expression in both cancer urothelial cell lines (Fig. 8A–C). Moreover, we also observed a significant decrease in the total amount of phosphorylated cortactin in muscle-invasive cancer urothelial cells T24 after the treatment with hAM homogenate (Fig. 8A,B). On the other hand, our study revealed that hAM extract had no effect on the expression of cortactin, RhoA, RhoC, and Cdc42, key proteins involved in reorganization of the actin cytoskeleton (Fig. 8A–C).

In normal urothelial NPU cells the effect of hAM homogenate and extract on the expression of proteins implicated in actin reorganization was opposite. We demonstrated that hAM preparations did not decrease the expression of the aforementioned proteins, but slightly increased the expression of cortactin. Interestingly, we were not able to detect any phosphorylated form of cortactin in NPU cells (Fig. 8A,D). Taken together, our results further confirm the notion that that hAM preparations do not have detrimental effect on normal urothelial cells.

### In muscle-invasive bladder cancer urothelial cells the combination of hAM homogenate and FAK inhibitors has greater anti-migratory effect than drug or hAM treatment alone

**Figure 9:**
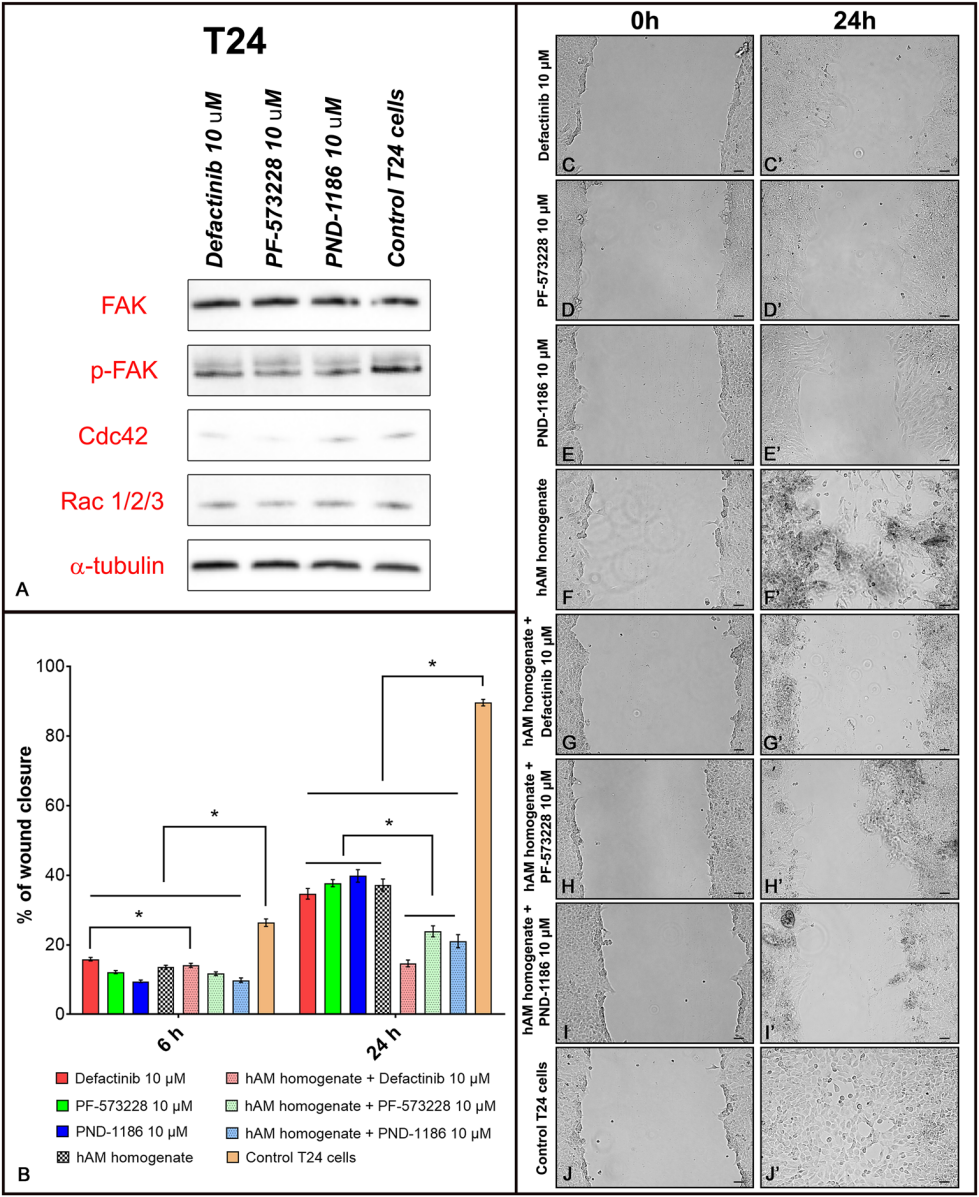
Combination of hAM homogenate and FAK inhibitors has greater anti-migratory effect than drug or hAM treatment alone. (**A**) Western blot analysis of FAK, p-FAK, Cdc42, Rac1/2/3 and α-tubulin protein expression after 24-h incubation of T24 cells with defactinib, PF-573228, PND-1186 or appropriate culture medium without FAK inhibitors (control samples). (**B**) The graph shows the percentage of mean wound closure ± standard error of the mean (SEM) in relation to the wound area at the beginning of the experiment (0 h). Data were obtained on the basis of three biological replicates. (**C**–**J′**) Representative images of wound healing of T24 cells treated with different FAK inhibitors, hAM homogenate, combination of FAK inhibitors and hAM homogenate and appropriate culture medium (control cells). The original blots are presented in Supplementary Fig. 2. The absence of full-length membranes is due to the cropping of the membranes prior to hybridization with primary antibodies. *P ≤ 0.05 vs. control group. Scale bars 50 µm.

As previous experiments have shown, the hAM preparation in the form of hAM homogenate had the greatest inhibitory effect on muscle-invasive bladder cancer urothelial cells. To determine whether hAM homogenate affects the same molecular targets as FAK inhibitors, T24 cells were treated with three different ATP-competitive FAK inhibitors (defactinib, PF-573228 and PND-1186). We showed that although the FAK inhibitors did not affect FAK expression, they inhibited the total amount of phosphorylated FAK (Fig. 9A). We also demonstrated that FAK inhibitors decreased the expression of proteins involved in actin cytoskeleton reorganisation, such as Cdc42 and Rac1/2/3, in the same way as hAM homogenate (Fig. 9A).

Next, we aimed to investigate the combined effect of hAM homogenate and FAK inhibitors on the migration of muscle-invasive cancer urothelial cells. We analysed the wound closure area of T24 cells after treatment with different FAK inhibitors (defactinib, PF -573,228, PND-1186), hAM homogenate, a combination of hAM homogenate and FAK inhibitors or culture medium containing neither hAM homogenate nor FAK inhibitors (control samples) (Fig. 9C–J′). In our experiments, we used a 10 μM concentration of FAK inhibitors determined on the basis of our previous studies (Supplementary Fig. S1) in which we had found that a 10 μM concentration of FAK inhibitors had a similar inhibitory effect on T24 cancer cell migration as hAM homogenate. We showed that FAK inhibitors and hAM homogenate inhibited T24 cancer cell migration at both analysed time points (6 and 24 h) (Fig. 9B, C′–J′). However, in our experimental settings we showed that FAK inhibitors in combination with hAM homogenate had the greatest anti-migratory effect on T24 muscle-invasive cancer urothelial cells at the 24-h time point (Fig. 9B,G–I′). Overall, we showed that FAK inhibitors in combination with hAM homogenate (Fig. 9B,G–I′) had greater anti-migratory effect than drug (Fig. 9B,C–E′) or hAM treatment alone (Fig. 9B,F–F′). We found that T24 cell migration was most effectively inhibited after combined treatment with hAM homogenate and defactinib (Fig. 9B, G–G′), followed by the combination of hAM homogenate and PND-1186 (Fig. 9B, H–H′) and the combination of hAM homogenate and PF-573228 (Fig. 9B, I–I′).

### hAM homogenate and extract moderately inhibit the expression of N-cadherin and MMP-2 in muscle-invasive bladder cancer urothelial cells

We then analysed whether the two hAM preparations affect the expression of epithelial-mesenchymal transition (EMT) markers in muscle-invasive cancer urothelial cells. To this end, after 24 h incubation of T24 cells with hAM homogenate, hAM extract or culture medium (control samples), the expression of N-cadherin and MMP-2 was analysed by qPCR, Western blotting and gelatin zymography.

**Figure 10:**
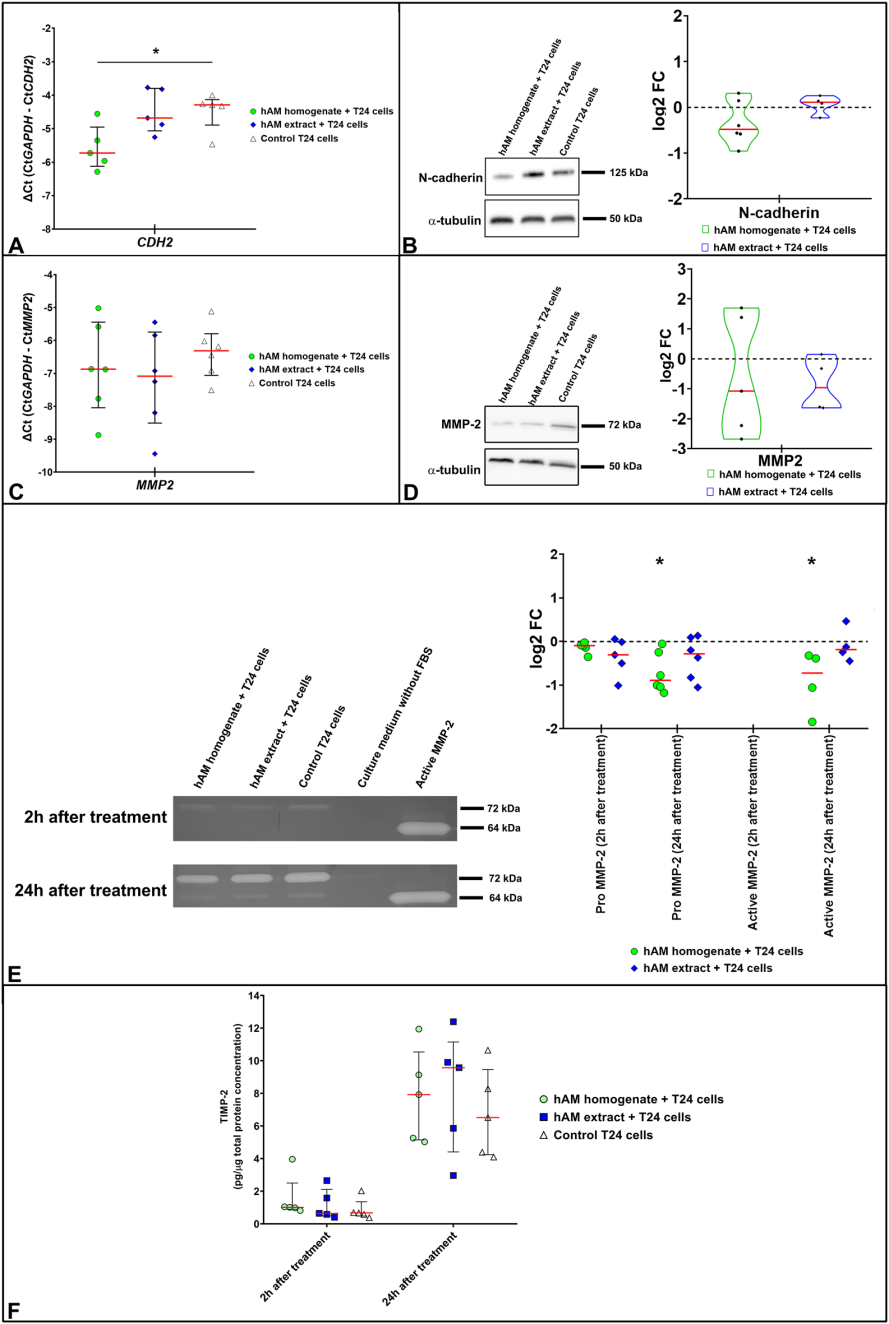
hAM homogenate and extract inhibit the expression of N-cadherin and MMP-2 in T24 cells. (**A**,**C**) Expression of CDH2 and MMP-2 in T24 treated with hAM homogenate, hAM extract or appropriate culture medium (control) without hAM preparations for 24 h. Expression of target genes was normalised to GAPDH levels. The qPCR results were presented as ΔCt (CtGAPDH − CtGOI). Each dot in the graphs A and C represents the value of one biological sample. The solid red line indicates the median, while the solid black lines represent the interquartile range. (**B**,**D**) The expression of N-cadherin and MMP-2 was quantified using Western Blot and normalised to the level of α-tubulin. Furthermore, the expression of the target proteins in the treated samples was normalised to the control samples (culture medium without hAM preparation) and these values are presented as log2 fold change (log2FC). The Western Blot results are shown as median (solid red line) with data range (minimum and maximum). Each point in the graphs represents the value of one biological sample. The dashed black lines represent the normalised value of the control sample (0). The most representative blot of the mean effect is presented. The original blots and gels are presented in Supplementary Fig. 2. The absence of full-length membranes is due to the cropping of the membranes prior to hybridization with primary antibodies. (**E**) Zymography analysis of the expression of pro- and active MMP-2 in the treated samples. The expression of target proteins was normalised to the control samples (culture medium) and these values were presented as log2 fold change (log2FC). Furthermore, the expression of pro-and active MMP-2 was normalised to the total protein levels. Each point in the graph represents the value of one biological sample. The solid red line indicates the median. The dashed black line represents the normalised value of the control sample (0). (**F**) The results of the ELISA tests were normalised to the total protein concentration and presented as such. Each point in the graph represents the value of one biological sample. The solid red line indicates the median. Results were obtained on the basis of 3–6 biological replicates for all experiments. *p ≤ 0.05.

Our study showed that *CDH2* gene expression was statistically significantly downregulated in T24 cells treated with hAM homogenate compared to control cells treated with culture medium (Fig. 10A). There was a slight decrease in *CDH2* expression in T24 cells treated with hAM extract, but the difference was not statistically significant compared to controls (Fig. 10A). At the protein level, we observed a similar trend in N-cadherin expression. Western blot results showed that N-cadherin expression was down-regulated in T24 cells treated with hAM homogenate compared to control T24 cells exposed to culture medium without hAM homogenate (Fig. 10B). In contrast, the hAM extract had no effect on N-cadherin expression (Fig. 10B).

We also showed that the hAM homogenate and extract decreased the expression of *MMP2*, but the difference in expression was not statistically significant compared to control cells (Fig. 10C). A similar downward trend was also observed at the protein level. Indeed, Western blot analysis showed that MMP-2 expression decreased in cells treated with hAM homogenate and hAM extract, but the difference in expression was not statistically significant compared to control T24 cells treated with culture medium without hAM preparations (Fig. 10D). Importantly, T24 cells treated with hAM homogenate or hAM extract secreted less pro-MMP-2 (72 kDa) than control T24 cells as early as 2 h after treatment (Fig. 10E). Furthermore, T24 cells secreted significantly less pro-MMP-2 after 24-h incubation with hAM homogenate in comparison to the control cells (Fig. 10E). Decreased levels of pro-MMP-2 were also observed in T24 cells treated with hAM extract, albeit to a lesser extent (Fig. 10E). On the other hand, we did not observe active MMP-2 (64 kDa) after 2 h treatment with different hAM preparations or with the corresponding culture medium (control samples). The active form of MMP-2 was only detected after a treatment period of 24 h. Our study showed that T24 cells treated with hAM homogenate, secreted significantly less active MMP-2 than control T24 cells. Although to a lesser extent, we also showed that T24 cells treated with hAM extract secreted less active MMP-2 than control T24 cells (Fig. 10E).

When we found that hAM preparations decreased MMP-2 expression and activity, we analysed whether this inhibitory effect was due to upregulated secretion of the tissue inhibitor of matrix metalloproteinase 2 (TIMP-2). Our study showed that T24 cells treated with hAM homogenate secreted slightly more TIMP-2 than T24 cells treated with hAM extract or culture medium (control samples) as early as 2 h after 24-h incubation with hAM homogenate (Fig. 10F). Furthermore, we were able to show that 24 h after the completed treatment with hAM preparations, the treated T24 cells secreted a greater amount of TIMP-2 than the control T24 cells treated with culture medium (Fig. 10F).

## Discussion

Growing body of evidence suggests that natural products may be a promising source for the development of novel anticancer drugs for bladder cancer treatment^26^. We have previously shown that preparations from natural biomaterial like hAM cause detachment of bladder cancer urothelial cells in a time-dependent manner. We also observed a decrease in their surface attachment ability and proliferation rate^22^. Therefore, the aim of the present study was to investigate the effect of hAM homogenate and extract on migration and invasion of bladder cancer urothelial cells and to elucidate the underlying mechanisms of action of hAM.

Migration and invasion are key processes involved in the metastatic spread of cancer cells to secondary sites^27^. In the present study, we demonstrated that hAM preparations, particularly hAM homogenate, impeded the migration of non-invasive and muscle-invasive cancer urothelial cells, as well as the invasion potential of the latter, at all experimental time points. The finding that the hAM preparations have an inhibitory effect on cancer cell invasion is consistent with the results of our previous study, in which we showed that hAM scaffolds inhibit the invasive potential of muscle-invasive T24 cells^18^. Our results are also in agreement with the findings of Jafari and colleagues, who showed that hAM-derived conditioned medium reduced the migration and invasion rate of MDA-MB-231 breast cancer cells^28^. On the other hand, our study showed that the effect of hAM preparations on the migration of normal urothelial cells was different from that of cancer urothelial cells. Our results differ slightly from those of other researchers who have used different normal cell lines and hAM preparations. Indeed, studies have shown that hAM patches promote the migration of human skin keratinocytes (HaCaT) and rabbit lung epithelial cells (Mv1Lu)^29–31^. Based on these results, we hypothesise that the effect on migration depends on the hAM preparation used, the type of normal cells, their degree of differentiation and the in vitro model used to study wound healing.

The hAM preparations not only reduced the migration rate of bladder cancer urothelial cells, but also influenced the morphology of the migrating cells and their migration pattern. The cells can migrate in two different ways, individually or collectively. Individual cell migration is characterised by the loss of intercellular contacts and the movement of cells one after the other. Collective migration differs from individual migration mainly in that migrating cells maintain intercellular contacts and remain connected during the migration process^32,33^. There is increasing evidence that cancer cells that migrate collectively are more aggressive and metastatic than those that migrate individually^34–36^. Our results show that muscle-invasive cancer urothelial cells treated with hAM homogenate or extract migrated individually and lost their directionality at the leading edge, while the non-treated cells migrated collectively. Thus, we concluded that hAM homogenate and extract converted the aggressive collective phenotype of T24 cells, typical of metastatic cells migrating in groups, to a less aggressive phenotype, typical of single migrating cancer cells. Moreover, we also showed that hAM homogenate specifically affects the migration of non-invasive transitional cell papilloma RT4 cells by reducing the formation of dynamic membrane protrusions responsible for driving the leading edge of the migrating cells (lamellipodia and filopodia)^37^. Collective cell migration is a complex cellular-biological process, during which epithelial cells migrate in groups and maintain a close intercellular connection^38,39^. Moreover, it is required for efficient wound healing of normal tissue.Our study has shown that hAM preparations do not affect the pattern and morphology of migrating normal urothelial cells as they maintain their collective epithelial migration pattern. Taken together, these results show that after treatment with hAM preparations, the wound healing processes of normal urothelial cells are not affected.

FAK contributes to the motility and invasiveness of normal and cancer cells by regulating various cell–matrix interactions^40–42^. Namely, FAK phosphorylates proteins and initiates various signalling pathways that regulate proliferation, survival, migration and invasion^43^. One of these signalling pathways is the PI3K/Akt/mTOR signalling cascade^7^, which is overexpressed and activated in several cancers, including bladder cancer^44^. It is known that the activated PI3K/Akt/mTOR pathway triggers bladder cancer cell migration and invasion^45,46^. Study from 2021 showed that inhibition of p-FAK by the FAK inhibitor (BI853520) reduced the expression of proteins that are part of the PI3K/Akt/mTOR pathway and subsequently reduced the invasive potential of ovarian cancer cells (SKOV3 cells and OVCAR3 cells)^47^. Furthermore, a recent study showed that the inhibition of FAK with homoharringtonine led to decrease in PI3K/Akt signalling cascade and consequently suppressed the migration of bladder cancer HT-1376 cells^48^. These findings are consistent with the results of our study, in which we showed that the hAM preparations, particularly the hAM homogenate, reduced the expression of FAK and p-FAK and inhibited the expression of the PI3K/Akt/mTOR signalling cascade in T24 muscle-invasive cancer urothelial cells. However, the hAM preparations had no effect on the PI3K/Akt/mTOR signalling pathway in non-invasive RT4 cells, although they inhibited FAK expression. In RT4 cells, we could not detect the phosphorylated form of Akt, which is required for its full catalytic activation. This result is consistent with the literature, in which various research groups have reported that p-Akt could not be detected in non-invasive urothelial papilloma RT4 cells^23–25^. Our results suggest that the effect of hAM preparations depends on the type of cancer cell. We hypothesise that the differences in effect are due to the fact that bladder cancer urothelial cells have different biological properties. Namely, the results of a recent study, in which 25 bladder cancer cell lines were whole-exome sequenced, showed that the T24 and RT4 cell lines have a different spectrum of mutations in genes that regulate various signalling pathways^49^.

Cytoskeletal remodelling is a crucial process required for cancer cell migration and invasion^50^. In response to specific extracellular signals, cortactin and small RhoGTPases (RhoA, RhoC, Cdc42 and Rac1) trigger key processes required for efficient cell migration, such as integrin activation, leading edge formation, focal adhesion recycling and trailing edge retraction^9,51–54^. Our study showed that hAM homogenate strongly reduced the expression of Cdc42 and Rac1/2/3 in non-invasive and invasive bladder cancer urothelial cells, while hAM extract had no inhibitory effect. Similarly, hAM homogenate reduced the expression of cortactin and its phosphorylated form. The fact that reduced expression or phosphorylation of cortactin leads to decreased migration is supported by several studies showing that inhibition of cortactin reduces cell motility^55,56^. On the other hand, hAM preparations did not inhibit the expression of cortactin and small RhoGTPases in normal porcine urothelial cells, but we could not detect a phosphorylated form of cortactin. We hypothesise that the absence of phosphorylated cortactin in NPU cells may be attributed to several factors. The first reason may be due to methodological limitations. The phosphorylated antibody (Cell Signaling Technology, Massachusetts, USA, catalog #4569) in our study specifically recognizes human epitopes, which could explain the lack of detection in porcine cells. Moreover, cortactin promotes lamellipodia and invadopodia formation, cell migration, and endocytosis^57,58^. In highly differentiated normal superficial urothelial cells, these processes are hindered, mainly due to the reorganization and lower expression of actin during differentiation of urothelial cells^59–63^. Nevertheless, further investigation is warranted to explore this phenomenon comprehensively..

Since we found that the preparation in the form of hAM homogenate had the greatest inhibitory effect on bladder cancer urothelial cells, we wanted to determine whether the mechanism of action of hAM homogenate is via downregulation of FAK expression and phosphorylation. To this end, we used ATP-competitive FAK inhibitors and investigated whether they affect similar molecular targets as hAM homogenate in T24 muscle-invasive cancer urothelial cells. We found that FAK inhibitors (defactinib, PF-573228 and PND-1186) only reduce the amount of the phosphorylated form of FAK, but do not affect its total concentration. Like hAM homogenate, FAK inhibitors also reduced the expression of proteins involved in actin cytoskeleton reorganisation, namely Cdc42 and Rac1/2/3. Based on these results, we hypothesised that hAM homogenate inhibits cancer cell migration similarly to FAK inhibitors. We demonstrated that both FAK inhibitors and hAM homogenate inhibited the migration of T24 cancer cells and that FAK inhibitors together with hAM homogenate had the strongest anti-migratory effect on T24 cells. The reason for the migration inhibitory effect may be due to a combined effect on FAK expression and phosphorylation. In contrast to FAK inhibitors, which only affect FAK phosphorylation, hAM homogenate also inhibits its expression. We hypothesise that both the inhibition of FAK expression by hAM homogenate and the reduced FAK phosphorylation resulting from the action of hAM homogenate and inhibitors strongly influence the expression and activity of a number of downstream effectors involved in the control of cell migration, including Cdc42 and Rac1/2/3.

In addition to FAK-related signalling, N-cadherin, a mesenchymal marker for epithelial-mesenchymal transition, plays a key role in cancer progression^64^. The increased expression of N-cadherin in cancer cells is associated with tumour aggressiveness^65^. Indeed, N-cadherin promotes collective cell migration and is thus involved in tumour spread to distant tissues^66–69^. Here we have shown that hAM homogenate strongly decreases the expression of N-cadherin at both the gene and protein levels. The finding that hAM homogenate reduces N-cadherin expression is consistent with the results of our previous study, in which we showed that hAM scaffolds reduce N-cadherin expression in T24 cells growing on these supports^18^. Taken together, our results suggest that hAM homogenate also reduces T24 cancer cell invasion by regulating N-cadherin expression. Furthermore, a decrease in N-cadherin expression triggers a switch from a collective to an individual mode of cell migration and consequently reduces the invasive/metastatic potential of T24 cancer cells.

Extracellular matrix degradation by MMPs is a key process required for cell invasion and metastasis^70^. MMPs belong to a family of zinc-dependent endopeptidases involved in the degradation of various cell adhesion molecules, thereby regulating intercellular and cell–cell interactions^71^. Cells synthesise and secrete MMPs in the form of inactive proenzymes (pro-MMPs), which are then activated in the extracellular space by proteolytic removal of the propeptide domain^72^. MMP expression correlates with tumour aggressiveness, disease stage and patient prognosis^73^. MMPs are involved in tumour cell migration, invasion and metastasis and promote cancer progression^72^. MMP-2 or gelatinase A is one of the MMPs commonly associated with bladder cancer^74^. The expression and activity of MMP-2 is increased in bladder cancer urothelial cells compared to normal tissues^75,76^. In the present study we showed that hAM homogenate and extract decreased the expression of MMP-2 at both gene and protein levels. Using gelatin zymography, we demonstrated that hAM preparations affected not only the intracellular concentration of MMP-2, but also the amount and activity of extracellular MMP-2. Reduced secretion of the active form of MMP-2 was also observed in T24 cells treated with hAM extract, but to a lesser extent. Thus, we conclude that preparations of hAM influence the invasion of T24 cancer cells by regulating MMP-2 expression and activity. The activity of MMP-2 is also regulated by tissue inhibitors of MMPs (TIMPs). In excess, TIMP-2 can bind to and inhibit MT1-MMP and both forms of MMP-2^77,78^. In contrast, TIMP-2 at low concentrations is essential for efficient MMP-2 activation^78^. TIMP-2 acts as a bridge between MT1-MMP and MMP-2 because it binds with its N-terminal domain to MT1-MMP and with its C-terminal domain to pro-MMP-2. This binding allows another neighbouring MT1-MMP, which does not have bound TIMP-2 molecule, to efficiently recruit the MMP-2 prodomain^72^. Due to the dual nature of TIMP-2, small shifts in the MMP-2/TIMP-2 balance can facilitate tumour progression and influence disease recurrence^78,79^. Here, we revealed that even though hAM homogenate increased TIMP-2 secretion from T24 cells 2 h after the end of treatment, the concentration of secreted TIMP-2 was relatively low compared to the TIMP-2 concentration at 24 h. This result suggests that activation of MMP-2 by the MT1-MMP/TIMP-2 complex occurs at a later time point, which is consistent with the results of gelatin zymography showing the presence of the active form only 24 h after the end of treatment. At the same time point (24 h after the end of treatment), we found that the preparations of hAM increased the amount of TIMP-2 secreted. From these results, we conclude that the increased secretion of TIMP-2 has an inhibitory function, as we detected less active MMP-2 in the T24 cells treated with hAM preparations, could be an additional anticancer mechanism of action of the hAM preparations.

Our study also showed that the anticancer effect also depends on the hAM preparation used. Namely, the hAM homogenate had a more detrimental effect of bladder cancer urothelial cells than hAM extract. From the results presented, we believe that the composition of hAM homogenate contributes to a greater anticancer effect. Our research group has previously shown that hAM homogenate is a mixture of hAECs, hAMSCs and tightly interwoven intercellular hAM fibers^22^. Moreover, hAM homogenate may contain various cellular contents (e.g., organelles and macromolecules) that are released from cells by homogenisation. On the other hand, the hAM extract was prepared by centrifuging the hAM homogenate at 1000×*g* and is free of hAM cells and ECM components. We hypothesise that the anticancer molecules may be trapped between the ECM proteins, as it has already been shown that hAM may act as a delivery tool of nanoparticles, antibiotics and drugs^80–84^. Furthermore, the gradual release of additional anticancer molecules from the remnants of hAM cells found in the hAM homogenate may contribute to the overall greater anticancer effect.

In conclusion, our study has contributed to a more comprehensive understanding of the signalling pathways involved in the anticancer activity of hAM. We have shown that hAM preparations, especially hAM homogenate, exert their anti-migration and anti-invasion effects on muscle-invasive bladder cancer urothelial cells by (1) down-regulating the FAK/PI3K/Akt/mTOR signalling pathway and (2) inhibiting the expression of proteins involved in actin cytoskeleton reorganisation and mesenchymal EMT markers such as N-cadherin and MMP-2, while simultaneously increasing the secretion of TIMP-2. On the other hand, we showed that the main mechanism of action of hAM preparations in non-invasive urothelial papilloma RT4 cells is via downregulation of FAK and p-FAK. In contrast to bladder cancer cells, we were able to demonstrate that neither hAM homogenate nor hAM extract have a damaging effect on bladder normal urothelial cells. Going further, hAM extract and homogenate must be first tested in animal studies and, if successful, be considered for clinical trials. Similarly to established bladder cancer treatments (e.g. Bacillus Calmette-Guerin, Mitomycin C, Doxorubicin), the hAM extract and homogenate could be applied as intravesical agents, which would add value as localised therapy has many potential benefits, including enhancing efficacy of treatment and reduced side effects^85,86^.

### Limitations of the study

1. This study focused on the effect of hAM preparations on the FAK/PI3K/Akt/mTOR signalling cascade in bladder cancer and normal urothelial cells in vitro. However, it needs to be investigated whether other, additional signalling pathways are also involved in the anti-cancer mechanism.
2. Further studies are needed to identify the molecules in hAM preparations that have an anti-cancer effect. In addition, future studies should aim to investigate the effect of hAM homogenate in combination with various drugs, such as cisplatin-based chemotherapeutics, which remain the gold standard for the treatment of bladder cancer.
3. Although we performed numerous independent experiments with different biological donors, each hAM homogenate and extract used was prepared from a single hAM. To address the potential biological variability, future research studies should be conducted with a pool of multiple biological samples of hAM.4. In vivo studies are needed to clarify the mechanism of action of hAM-related preparations and their safety profile in the living organisms. Currently, our research group is conducting a long-term in vivo study using an N-butyl-N-(4-hydroxybutyl) nitrosamine (BBN) to induce early bladder carcinogenesis in mice that resembles human bladder carcinogenesis in its morphological, biological, and molecular features. Our aim is to further explore the anti-cancer effect of hAM homogenate in such an in vivo setting.

## Methods

### Normal and cancer urothelial models

Human muscle-invasive bladder cancer T24 cells and human non-invasive transitional cell papilloma RT4 cells were purchased from the American Type Culture Collection (ATCC) and grown in culture medium consisting of equal parts A-DMEM (Gibco, Life Technologies, Thermo Fisher Scientific, Waltham, MA, USA) and F12 (Sigma-Aldrich, Merck, Darmstadt, Germany), 5% foetal bovine serum (FBS; Invitrogen, Carlsbad, CA, USA) and 4 mM glutamax (Gibco, Thermo Fisher Scientific, Waltham, MA, USA).

The T24-eGFP cells were prepared by monoclonal selection of T24 cells transduced with third-generation lentiviral particles carrying the enhanced green fluorescent protein (eGFP) under the phosphoglycerate kinase (PGK) promoter and the puromycin resistance gene for the selection of transduced cells. Lentiviral particles were prepared by transfection of packaging 293T cells (ATCC CRL-3216TM) with the transfer plasmid pLenti PGK GFP Puro (w509-5) (Addgene plasmid #19070)^87^, packaging plasmids pMDLg/pRRE and pMD2.G (Addgene plasmids #12251 and #12259), and the envelope plasmid pRSV-Rev (Addgene plasmid #12253)^88^, as previously described^89^. Culture medium containing viral particles was harvested from the transfected cells at 48 and 72 h post-transfection and concentrated lentiviral particles were prepared using Lenti-X™ Concentrator (Tekara, Shiga, Japan) according to the manufacturer’s instructions. For the transduction, 200 µl of concentrated particles were added to 1 × 10^5^ T24 cells plated in a well of a 6-well plate in the presence of 8 µg/ml of polybrene (Sigma-Aldrich, St. Louis, Mo, USA). After 24 h, the medium was replaced with a fresh medium, and after 48 h, 2 µg/mL of puromycin (Sigma-Aldrich, St. Louis, Mo, USA) was added to the medium. After 1 week of culturing under the selective pressure of puromycin, the cells were plated in a 96-well plate at a concentration of ~ 1 cell per well to prepare a monoclonal cell line. When the cells formed colonies (~ 2 weeks), wells with one colony per well were inspected using fluorescence microscopy (inverted fluorescence microscope IX70; Olympus, Tokyo, Japan) and a clone with a uniform eGFP fluorescence intensity was selected for further propagation. The stably transduced T24-eGFP cells were grown in the same culture medium as T24 cells.

Normal porcine urothelial cells (NPU) were established as previously described^62,63,90–94^ and grown in culture medium containing equal amounts of MCDB153 (Sigma-Aldrich, St. Louis, MO, USA) and A-DMEM (Gibco, Life Technologies, Thermo Fisher Scientific, Waltham, MA, USA) supplemented with 15 μg/ml adenine (Sigma-Aldrich, St. Louis, MO, USA), 0.1 mM phosphoethanolamine (Sigma-Aldrich, St. Louis, MO, USA), 5 μg/ml insulin (Sigma-Aldrich, St. Louis, MO, USA), 0.5 μg/ml hydrocortisone (Sigma-Aldrich, St. Louis, MO, USA), 2 mM glutamax (Gibco, Thermo Fisher Scientific, Waltham, MA, USA), and 2.5% FBS (Invitrogen, Carlsbad, CA, USA). After reaching confluence, NPU cells were cultured for another 3 weeks in the same culture medium as described above, but without FBS and with 2.5 mM CaCl_2_. Normal and cancer urothelial in vitro models were cultured at 37 °C and 5% CO_2_. All urothelial models were routinely tested for mycoplasma infection using the (MycoAlert^®^ Mycoplasma Detection Kit, Lonza, Cologne, Germany) and were mycoplasma free.

The preparation of primary urothelial cells from porcine urinary bladders was approved by the Veterinary Administration of the Slovenian Ministry of Agriculture and Forestry in accordance with the Animal Health Protection Act and the Instructions for Granting Permits for Animal Experiments for Scientific Purposes (Decree No. U34453-15/2013/2).

### Preparation of human amniotic membrane homogenate and extract

Each donor was informed about the study and signed a written informed consent form. This study was approved by the National Medical Ethics Committee of the Republic of Slovenia (Decree No. 43/12/09 and 0120-179/2018/5). The hAM homogenate was prepared as previously described^22^. Briefly, after elective caesarean section, the hAM was separated from the human chorionic membrane (hCM), washed with sterile phosphate-buffered saline (PBS) and cut into small pieces (3 × 3 cm). Three parts of appropriate culture medium without FBS or CaCl_2_ were added to one part of the hAM pieces (dilution ratio 1:3). After homogenisation, the prepared homogenates were filtered through sterile nylon membrane with a pore size of approximately < 1 mm and stored at – 80 °C until further use. To prepare the hAM extract, the hAM homogenate was thawed and centrifuged at 1000×*g* for 10 min at room temperature. After centrifugation, the pellet containing the extracellular matrix (ECM) proteins, nuclei, dead cells and cell debris was discarded and the clear supernatant, which we termed hAM extract, was immediately used for the experiments. For the wound healing and transwell invasion assays, we diluted the original hAM homogenate and extract by half in an appropriate culture medium and named the so-prepared preparations as twofold diluted hAM homogenate and twofold diluted hAM extract. Before each experiment (with the exception of transwell invasion assay), the hAM homogenates and hAM extracts were supplemented with 5% FBS for the treatment of T24 and RT4 cells or 2.5 mM CaCl_2_ for the treatment of NPU cells.

The studies involving human participants were reviewed and approved by the National Medical Ethics Committee of the Republic of Slovenia and all participants provided their written informed consent to participate in this study. Procedure for preparation of amniotic membrane homogenate is part of patent application: ERDANI-KREFT, Mateja, ŽELEZNIK RAMUTA, Taja. Procedure for preparation of amniotic membrane homogenate, based antimicrobial agent EP 3 917 549 A0, 2021-12-08. Munich: European Patent Office, 2021^95^.

### Wound healing assay

Wound healing assay was used to evaluate the influence of hAM preparations on migration of cancer and normal urothelial cells in specific time points. T24, RT4 and NPU cells were seeded in 12-well plates or Ibidi Culture-Insert 4 well µ-dish and grown to confluence. Cells were cultured in serum-free culture medium 20–24 h prior to treatment. The cell-free gap was formed by a 200 µl pipette tip or by removing the culture insert. Cells were treated with the appropriate culture medium (control) or the indicated hAM preparations. Approximately, 10–15 images of scratched areas per well were acquired after illumination with a DAPI filter block (λ = 330 − 380 nm) using an Eclipse E300 inverted phase contrast fluorescence microscope equipped with CFI Plan Fluor 10× objective with NA 0.30 (Nikon, Tokyo, Japan) at time zero and/or after 6, 24, 48 or 96 h and analysed using ImageJ software^96^. The data are representative of at least 5 independent experiments and are presented as a percentage of wound closure, calculated according to the following formula:

$$\% \; of \; wound \; closure=\frac{(\mathrm{Area \; t}=0 \; \mathrm{ hr})-(\mathrm{Area \; t}=\mathrm{\Delta \; hrs})}{(\mathrm{Area \; t}=0 \; \mathrm{ hr})} \times 100 \%$$

### Live-cell imaging

For live cell imaging, T24-eGFP, RT4 and NPU cells were seeded on Ibidi Culture-Insert 4 well µ-dish and grown to confluence. To create a cell-free gap, the culture inserts were removed and the non-adherent cells were washed with PBS. Cells were incubated in the appropriate culture medium (control) or the indicated hAM preparations for up to 24 h. Time-lapse fluorescence and/or phase contrast images of multiple microscopic fields were acquired using a LSM 900 confocal microscope equipped with Plan-Apochromat 20× objective with NA 0.8. (Zeiss, Germany) every 15 min. Diode laser 488 nm (25-mW) was used for excitation of eGFP. Emission was detected between 502 and 543 nm. Images were analyzed with ZEISS ZEN lite 3.2.

### Transwell invasion assay

For the invasion assay, transwell chambers with membranes of 8 μm pore size were coated with diluted Matrigel matrix (CORNING) for 1 h at 37 °C. After gelation, the upper transwell chamber was washed and 5.6 × 10^4^ T24 cells were added to 0.5 ml serum-free culture medium (controls), serum-free hAM homogenate or serum-free hAM extract. The lower chamber was filled with 1.5 ml culture medium supplemented with chemoattractant (10% FBS). After incubation at 37 °C and 5% CO_2_ for 24 h, T24 cells were fixed in 4% PFA/PBS, washed with sterile PBS and stained with Hoechst in PBS (10 µg/ml) for 15 min. Eight randomly selected fields per well in the upper chamber were imaged with an Eclipse E300 fluorescence microscope and the total number of cells in the invasion assay was calculated. Subsequently, the non-invaded cells were removed from the upper surface of the transwell insert with a cotton swab and 8 randomly selected fields per well with the remaining invaded cells on the lower surface were imaged and counted. The results are representative of at least 4 independent experiments and are presented as a percentage of invaded cells (number of invaded cells/total number of cells × 100%).

### Western Blot analysis

Confluent cultures of T24, RT4 and NPU cells were treated with hAM homogenate, hAM extract or the appropriate culture medium (control) for 24 h. After treatment, cells were washed with sterile PBS and lysed in ice-cold RIPA buffer (Merck, Kenilworth, NJ, USA) with a cocktail of phosphatase and protease inhibitors (Thermo Fisher Scientific, Waltham, MA, USA). The Pierce BCA Protein Assay Kit (Thermo Fisher Scientific, Waltham, MA, USA) was used to determine the total protein concentration in the samples. 50 µg of proteins was separated using the 4–20% and 6% Novex Wedge Well Tris–Glycine gels (Invitrogen, Carlsbad, CA, USA). After electrophoresis, the gels were transferred to nitrocellulose membranes (Amersham), which were blocked in a solution containing 5% non-fat milk and 0.1% Tris-Buffered Saline/Tween 20 (TBS-T) for 2 h at RT and incubated with primary antibodies at 4 °C (Supplementary Table 1). The next day, membranes were washed in TBS-T and incubated with an appropriate HRP-conjugated secondary antibody for 1.5 h. Visualisation of protein bands by chemiluminescence was performed using SuperSignal West Pico chemiluminescent substrate (Thermo Scientific, Waltham, MA, USA). Densitometric analysis was performed using ImageJ software. The α-tubulin was used as a loading control. Western blot data are representative of 3–6 independent experiments and are expressed as log2 fold change in expression compared to the control samples. The original blots presented in Figs. 6, 7, 8, 9, 10 are available in Supplementary Fig. 2. The absence of full-length membranes is in some cases due to the cropping of the membranes prior to hybridization with primary antibodies. Supplementary Fig. 3. contains images of visible membrane edge after adjusting the contrast, whereas Supplementary Fig. 4 contains multiple exposure images of membranes with high contrast. In addition, the original, unedited blots for all biological replicates can be found in Supplementary Fig. 5. Supplementary Fig. 6 contains original blots with molecular size markings from multiple independent experiments used to demonstrate the target antigen specificity.

### Quantitative real-time PCR

Confluent cultures of T24, RT4 and NPU cells were treated with hAM homogenate, hAM extract or the appropriate culture medium (control) for 24 h. After treatment, cells were washed with sterile PBS and total RNA was isolated using the QuickRNA mini prep kit (Zymo Research, Irvine, CA) according to the manufacturer's protocol. Total RNA concentration was quantified using the Qubit Flex Fluorometer (Thermo Fisher Scientific, Waltham, Massachusetts, USA) and RNA was converted to cDNA using the Reverse Transcription System (Promega) according to the manufacturer's protocol. Real-time PCR (RT-PCR) was performed using the MIC qPCR Cycler (Bio molecular systems). Each reaction mix contained 5 µL FAST SYBR Green Master Mix (Thermo Fisher Scientific, Waltham, Massachusetts, USA), 2.5 µL 200 nM specific forward and reverse primers, 0.5 µL dH_2_O and 2 µL cDNA (0.5 ng/mL). The primers used in this study were designed with NIH Primer- BLAST and are listed in Supplementary Table 2. The PCR cycle parameters were: 95 °C for 20 s, followed by 40 cycles at 95 °C for 3 s and 60 °C for 30 s. The samples were run in triplicates. In all experiments, *GAPDH* was used as an endogenous control for normalisation. Data are representative of 3–6 independent experiments and are presented as ΔCt, i.e., the difference between the Ct values of the reference gene and the gene of interest (ΔCt reference gene − ΔCt gene of interest).

### ELISA

Confluent cultures of T24 cells were treated with hAM homogenate, hAM extract or the appropriate culture medium (controls) for 24 h. After treatment, cells were rinsed with PBS and incubated with the appropriate culture medium without FBS. The culture medium was collected 2 and 24 h after the treatment period. Total protein concentration in the samples was determined using the Pierce BCA Protein Assay Kit (Thermo Fisher Scientific, Waltham, MA, USA). The concentration of TIMP-2 protein in the conditioned medium was analysed using the TIMP-2 DuoSet Elisa Kit (R&D Systems, Minneapolis, USA) according to the manufacturer's protocol. TIMP2 levels were measured and normalised to total protein levels. Data are representative of at least five independent experiments.

### Gelatin zymography

The expression and the activity of matrix metalloproteinase-2 (MMP-2) was analysed using gelatin zymography in the same culture medium samples as reported in the previous section. Briefly, 5 µg of proteins was separated using homemade 10% SD-polyacrylamide electrophoresis gels (SDS-PAGE) containing 0.1% gelatine at 125V and 4 °C. To identify the active form, recombinant MMP-2 protein (ab81550, Abcam) was applied to the same gels at a concentration of 5 ng/µl. After electrophoresis, the gels were washed with distilled water and incubated twice in a succession in renaturation buffer (2.5% Triton X-100 in distilled water) for 30 min at room temperature. The gels were then rinsed with distilled water and incubated in developing buffer (0.5 M Tris HCl pH = 7.8, 2M NaCl, 0.05M CaCl_2_ and 0.2% Triton X-100 in distilled water), first for 30 min at room temperature and then for 22 h at 37 °C. The gels were stained in a staining solution (0.5% Coomassie Brilliant Blue R-250, 5% methanol, 10% acetic acid in distilled water) for one hour and immediately decoloured in a solution of 5% ethanol, 10% acetic acid and distilled water. Images of the gels were scanned using an Epson perfection 4990 Photo Scanner (Epson America Inc., CA, USA). Densitometric analysis of gelatinolytic activity bands was performed using ImageJ software. Total protein measurement was used for loading control. Gelatin zymography data are representative of 4–6 independent experiments and are expressed as log2 fold change in expression compared to the control samples.

### Quantification and statistical analysis

All statistical analyses were performed using GraphPad Prism 8 software. Normally distributed data were presented as mean ± standard error of the mean (SEM), while non-normally distributed data were presented as median and interquartile range. To compare statistical differences between at least 3 experimental groups, a parametric one-way analysis of variance (ANOVA) followed by Tukey's post hoc test or the non-parametric Kruskal–Wallis test with Dunn's correction for multiple comparisons was used. A p-value of < 0.05 was considered statistically significant.

### Supplementary Information

Supplementary Information.

Supplementary Video 1.

Supplementary Video 2.

Supplementary Video 3.

## Data Availability

Any additional information required to reanalyze the data reported in this paper is available from the corresponding author (M.E.K) upon request.
