# Supplementary material for: Human amniotic membrane inhibits migration and invasion of muscle-invasive bladder cancer urothelial cells by downregulating the FAK/PI3K/Akt/mTOR signalling pathway
Source: Sci Rep. 2023 Nov 6;13:19227. doi: 10.1038/s41598-023-46091-2 (PMC10628262; doi:10.1038/s41598-023-46091-2)
Supplement: Supplementary file 1 — Supplementary Information. [file 41598_2023_46091_MOESM1_ESM.docx]

# Supporting information

**Human amniotic membrane inhibits migration and invasion of muscle-invasive bladder cancer urothelial cells by downregulating the FAK/PI3K/Akt/mTOR signalling pathway**

Aleksandar Janev^1^, Taja Železnik Ramuta^1^, Urška Dragin Jerman^1^, Hristina Obradović^1^, Urška Kamenšek^2^, Maja Čemažar ^2^, Mateja Erdani Kreft^1^*

^1^Institute of Cell Biology, Faculty of Medicine, University of Ljubljana, Ljubljana, Slovenia

^2^Department of Experimental Oncology, Institute of Oncology Ljubljana, Ljubljana, Slovenia

* Corresponding author

E-mail: mateja.erdani@mf.uni-lj.si

**Supplementary Table 1.** Primary antibodies used for Western blot analysis.

| Antibody | Host | Dilution | Supplier |
| --- | --- | --- | --- |
| FAK | Rabbit | 1:1000 | Cat. No. 3285; Cell Signaling Technology, Inc. |
| p-FAK | Rabbit | 1:1000 | Cat. No. 70-025-5 Clone: 31H5L17; Invitrogen™ |
| PI3K p110α | Rabbit | 1:1000 | Cat. No. 4249; Cell Signaling Technology |
| Total Akt | Mouse | 1:2000 | Cat. No. 2920; Cell Signaling Technology |
| p-Akt | Rabbit | 1:2000 | Cat. No. 4060; Cell Signaling Technology |
| mTOR | Rabbit | 1:1000 | Cat. No. 2983; Cell Signaling Technology |
| p-mTOR | Rabbit | 1:1000 | Cat. No. 2971; Cell Signaling Technology |
| α-tubulin | Mouse | 1:2000 | Cat. No. T6199; Sigma-Aldrich |
| Cortactin | Rabbit | 1:1000 | Cat. No. 3503; Cell Signaling Technology |
| p-cortactin | Rabbit | 1:1000 | Cat. No. 4569; Cell Signaling Technology |
| RhoA | Rabbit | 1:1000 | Cat. No. 2117; Cell Signaling Technology |
| RhoC | Rabbit | 1:1000 | Cat. No. 3430; Cell Signaling Technology |
| Cdc42 | Rabbit | 1:1000 | Cat. No. 2466; Cell Signaling Technology |
| Rac 1/2/3 | Rabbit | 1:1000 | Cat. No. 2465; Cell Signaling Technology |
| N-cadherin | Rabbit | 1:1000 | Cat. No. ab18203; Abcam |
| MMP-2 | Rabbit | 1:1000 | Cat. No. 40994; Cell Signaling Technology |

**Supplementary Table 2.** Primers used for qPCR.

| Gene  name | Species | Forward primer (5'-3') | Reverse primer (5'-3') |
| --- | --- | --- | --- |
| *PTK2* | *Human* | CATGCCCTCAACCAGGGATT | CACGCTGTCCGAAGTACAGT |
|  | *Sus scrofa* | GCAGTCCGAGTTCTCCTTGA | TTTGGGTCAAGGTAAGCAGC |
| *PIK3CA* | *Human* | AGAGCCCCGAGCGTTT | TCGTGGAGGCATTGTTCTGA |
|  | *Sus scrofa* | CCCAGGTGGAATGAATGGCT | GCCAATGGACAGTGTTCCTCT |
| *AKT1* | *Human* | ATTTCCCTCTTTGGAGGCTGTG | CAGCCAACCCTCCTTCACAAT |
|  | *Sus scrofa* | CTGCACAAACGAGGCGAGT | CGCTCCTTGTAGCCGATGAA |
| *MTOR* | *Human* | GAATGCCACCCGAATTGGC | AATTCCACGTACTCAGCGGT |
|  | *Sus scrofa* | TACGACCACCTGACCCTGAT | CGATCAAACCACACCTCGGA |
| *CDH2* | *Human* | AACAGCAACGACGGGTTAGT | CAGACACGGTTGCAGTTGAC |
| *MMP2* | *Human* | GACCAGAATACCATCGAGACCA | GTGTAGCCAATGATCCTGTATGTG |
| *GAPDH* | *Human* | GAAGGTCGGAGTCAACGGAT | TTCCCGTTCTCAGCCATGTAG |
|  | *Sus scrofa* | TCGGAGTGAACGGATTTGGC | TGCCGTGGGTGGAATCATAC |


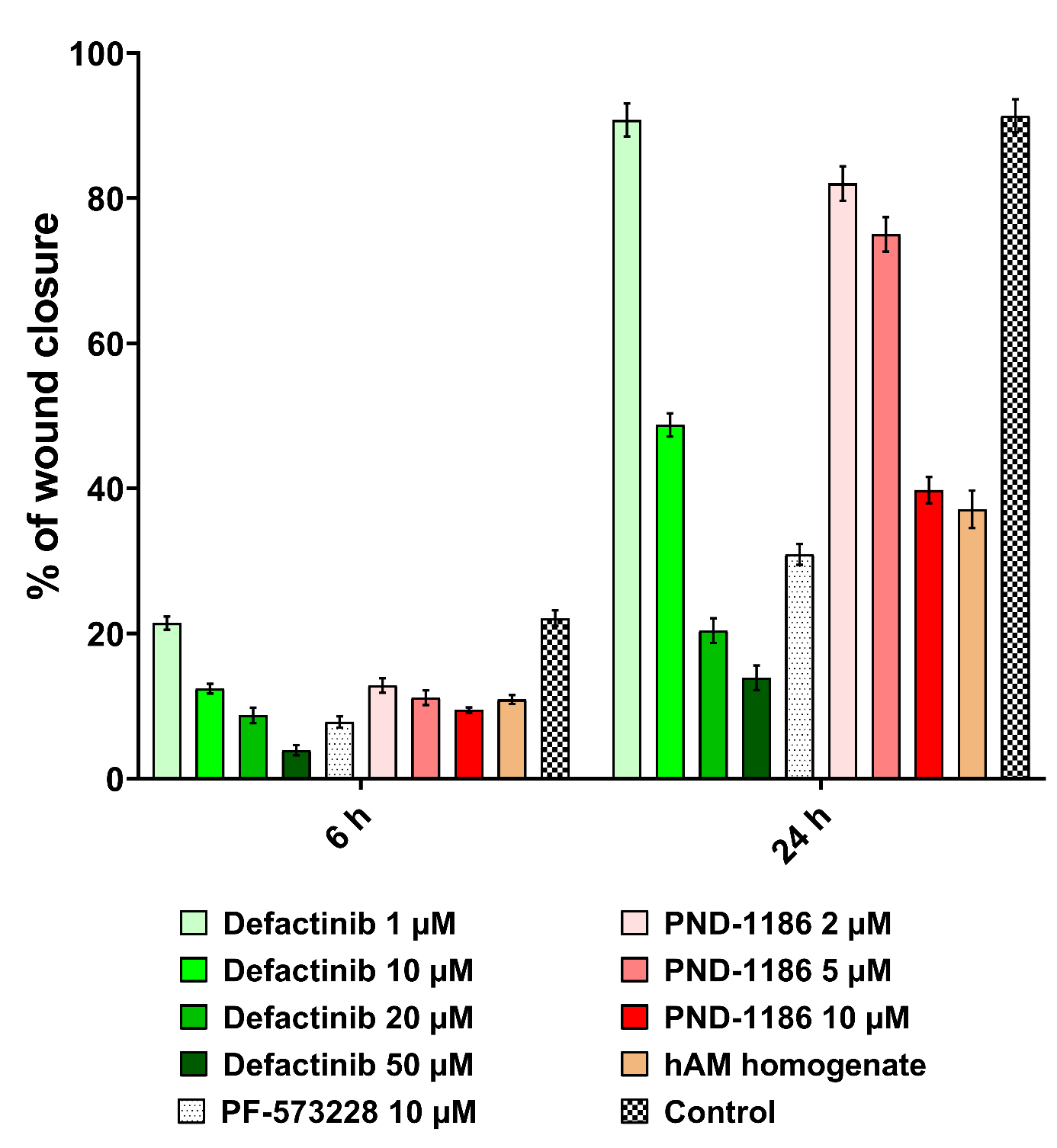


Supplementary Figure 1. **FAK inhibitors at 10 μM concentration have similar inhibitory trend as hAM homogenate.** Percentage of wound healing of T24 cells after treatment with different concentration of FAK inhibitors, hAM homogenate or appropriate culture medium (control samples). Data are presented as mean ± standard error of the mean (SEM) of at least three independent experiments.

Supplementary Movie S1. **Time-lapse imaging of T24 cells treated with hAM homogenate, hAM extract and culture medium (controls).** Movie length 22 hours, pictures are taken every 15 minutes. https://www.dropbox.com/s/5zhb8x9f0nhjlju/Supplementary%20movie%201.mp4?dl=0

Supplementary Movie S2. **Time-lapse imaging of RT4 cells treated with hAM homogenate, hAM extract and culture medium (controls).** Movie length 24 hours, pictures are taken every 15 minutes. https://www.dropbox.com/s/5jq978vo550kgci/Supplementary%20movie%202.mp4?dl=0

Supplementary Movie S3. **Time-lapse imaging of NPU cells treated with hAM homogenate, hAM extract and culture medium (controls).** Movie length 11 hours, pictures are taken every 15 minutes. https://www.dropbox.com/s/jcwry2297abrz4b/Supplementary%20movie%203.mp4?dl=0

# Supplementary Figure 2. Full unedited images of blots and gels

##### Cells T24

FAK

p-FAK

Alpha-tubulin


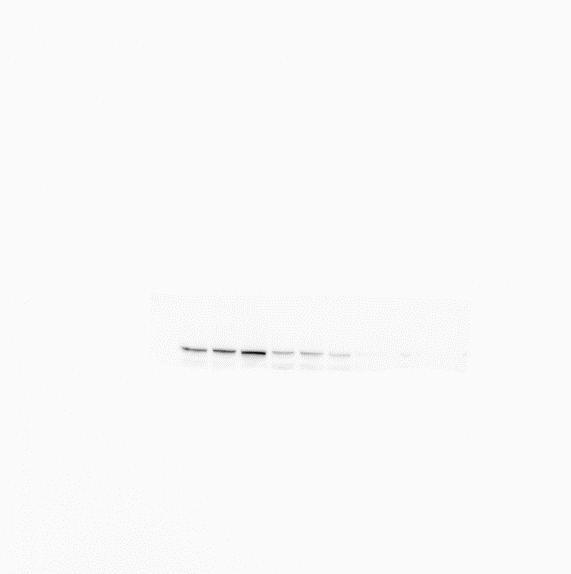


*120 kDa*

1 2 3


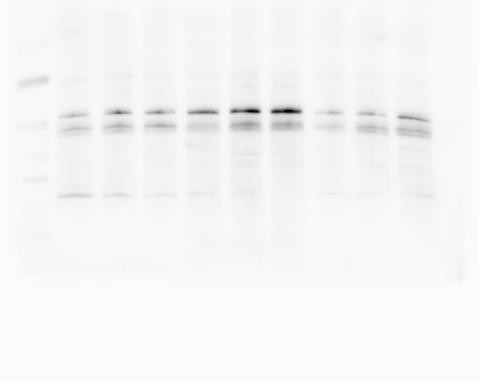


1 2 3

*50 kDa*


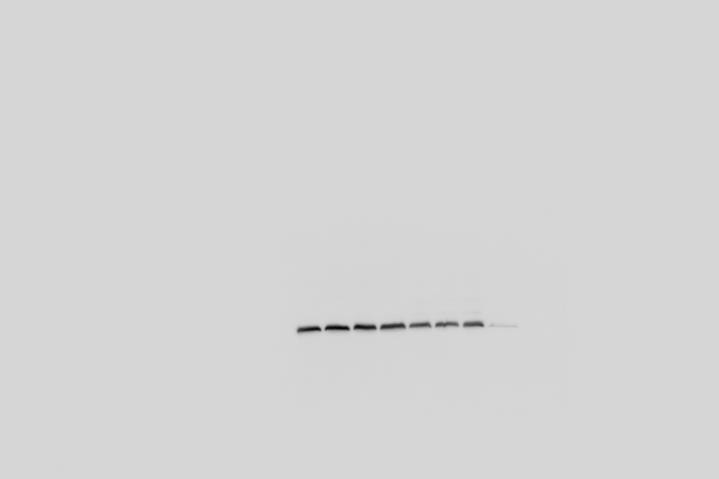


1 2 3

*50 kDa*

##### Cells RT4

FAK p-FAK Alpha-tubulin


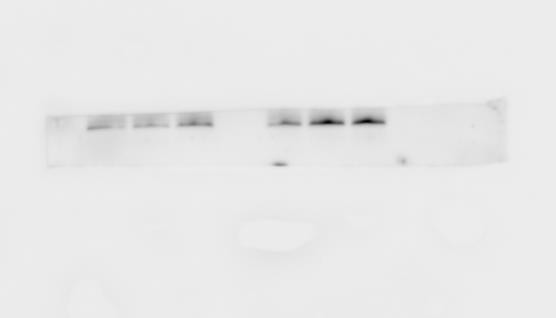


1 2 3

*120 kDa*


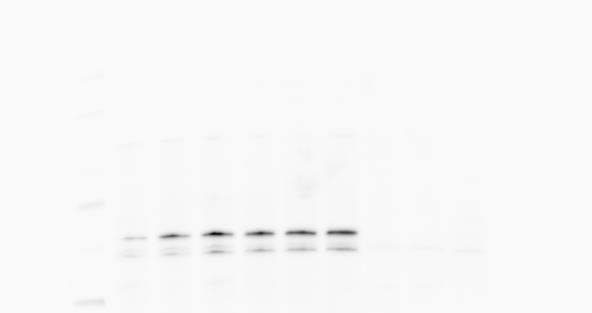


1 2 3

*50 kDa*


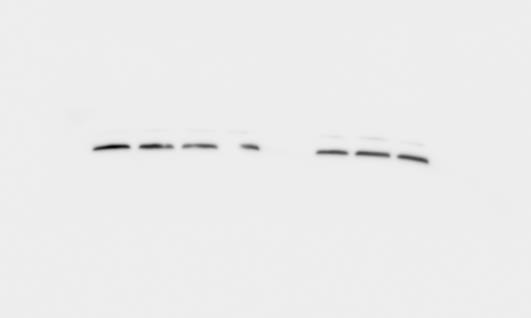


1 2 3

*50 kDa*

FAK

##### Cells NPU

p-FAK


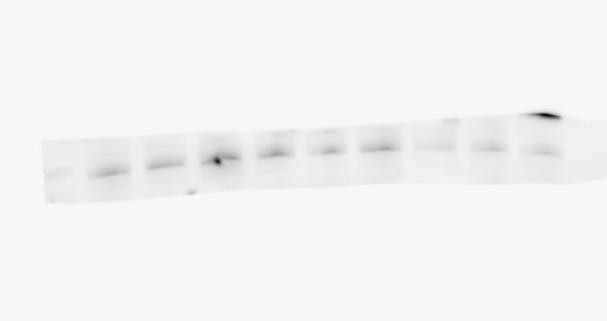


1 2 3

*50 kDa*

Alpha-tubulin


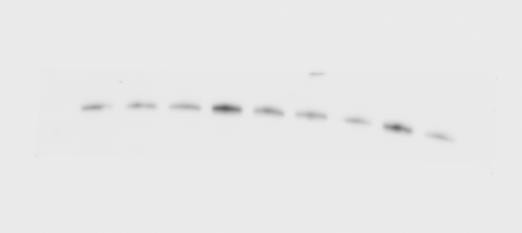


1 2 3

*50 kDa*


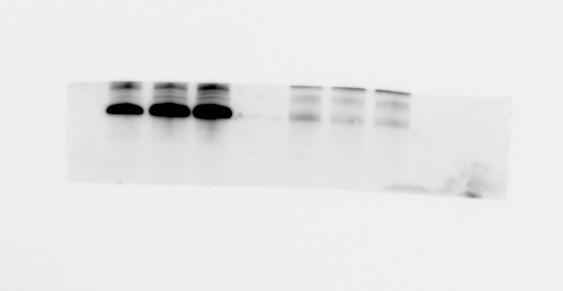


*125 kDa*

1 2 3

Pi3K p110a

##### Cells T24

Akt


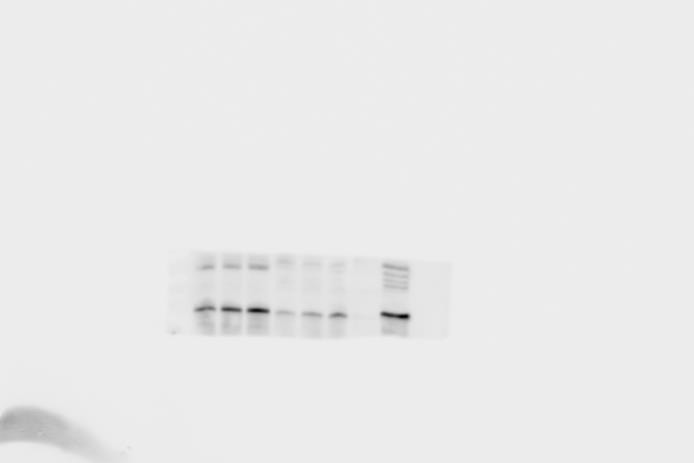


*110 kDa*

1 2 3

p-Akt


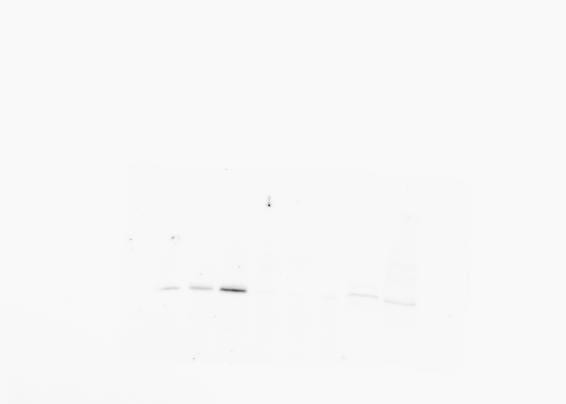


*60 kDa*

1 2 3


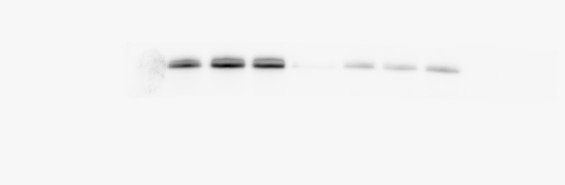


*60 kDa*

1 2 3


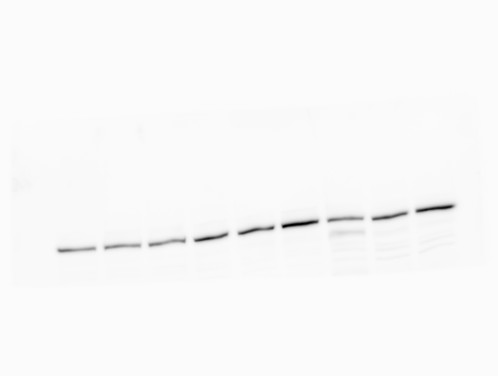

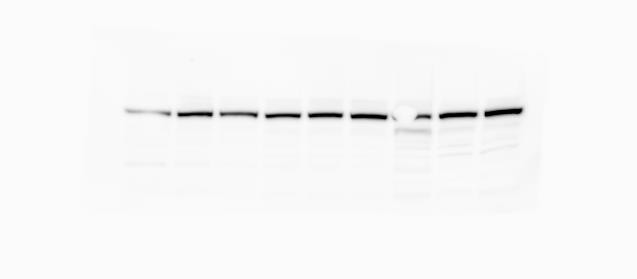


mTOR

p-mTOR

1 2 3

*289 kDa*

1 2 3

*289 kDa*


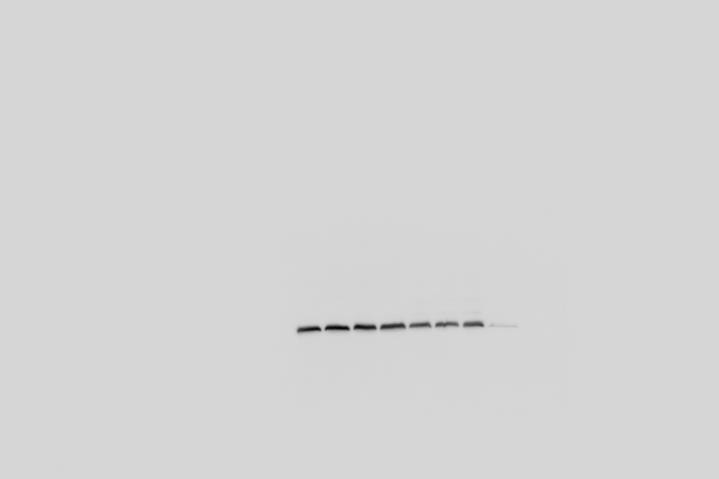


Alpha-tubulin

*50 kDa*

1 2 3

Pi3K p110a

##### Cells RT4

Akt

p-Akt


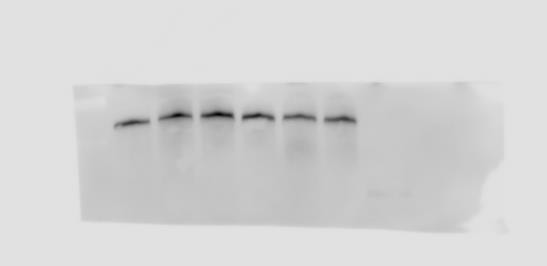


1 2 3

*110 kDa*


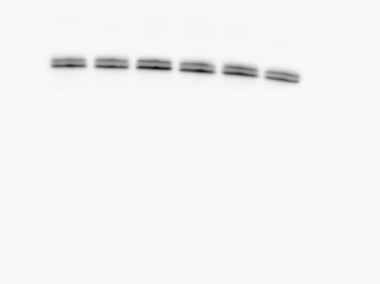


1 2 3

*60 kDa*


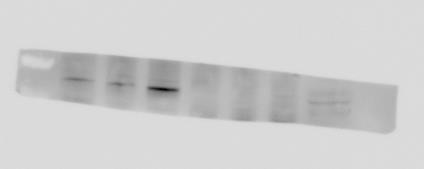


1 2 3

*60 kDa*

mTOR


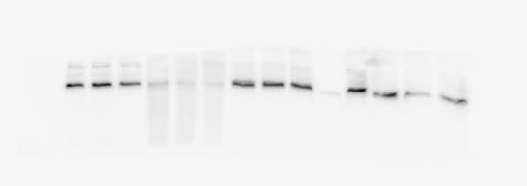


1 2 3

*289 kDa*

p-mTOR

Alpha-tubulin


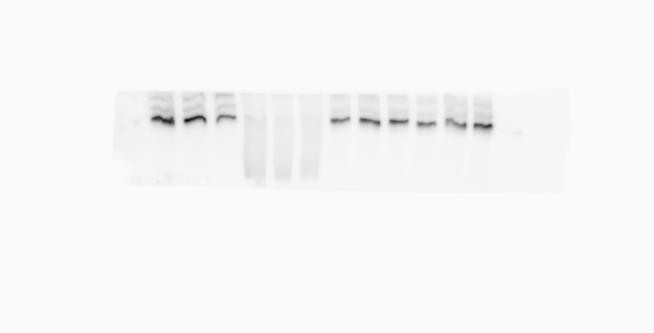


1 2 3

*289 kDa*


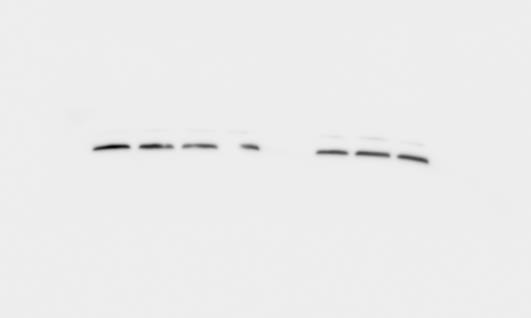


1 2 3

*50 kDa*

##### Cells NPU

Pi3K p110a

Akt

- 1. kt


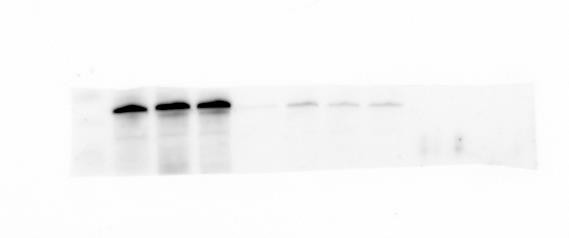


1 2 3

*110 kDa*


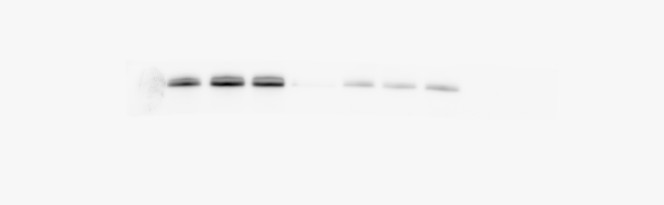


1 2 3

*60 kDa*


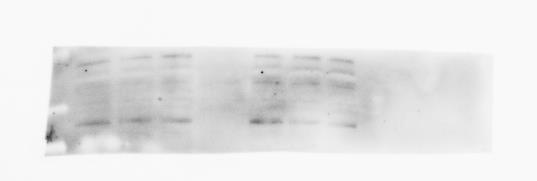


1 2 3

*60 kDa*


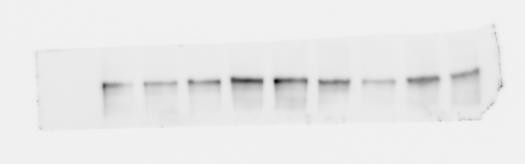


mTOR

1 2 3

*289 kDa*


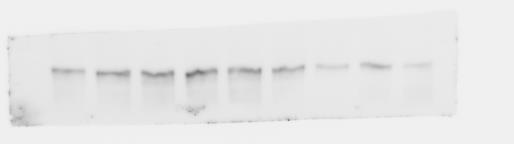


p-mTOR 1 2 3

*289 kDa*


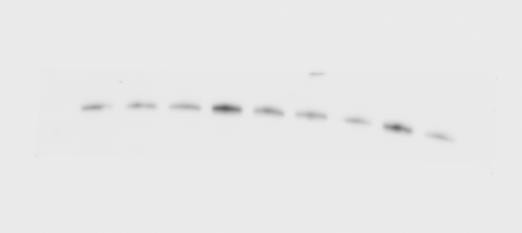


Alpha-tubulin

1 2 3

*50 kDa*

##### Cells T24

p-cortactin


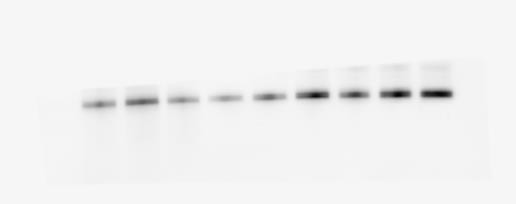


cortactin

1 2 3

*85 kDa*

RhoA

Rac1/2/3


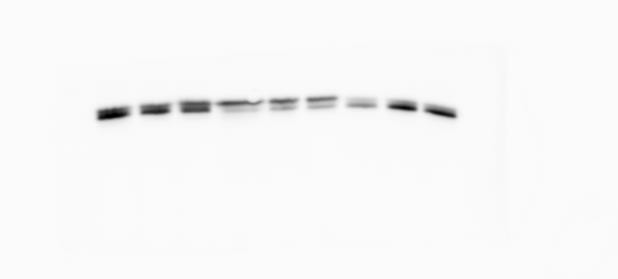


1 2 3

*21 kDa*


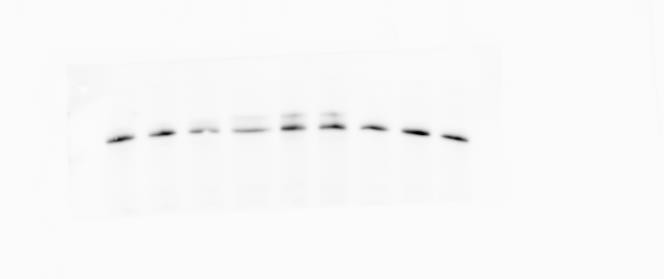


1 2 3

*21 kDa*


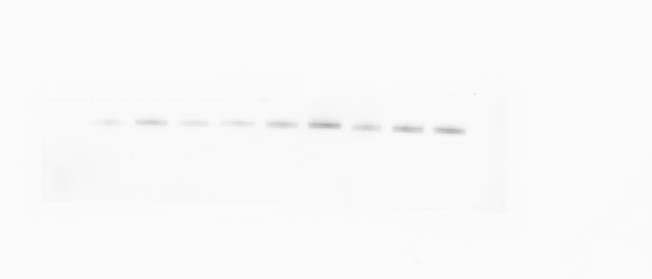


1 2 3

*85 kDa*

RhoC

Cdc42

Alpha-tubulin


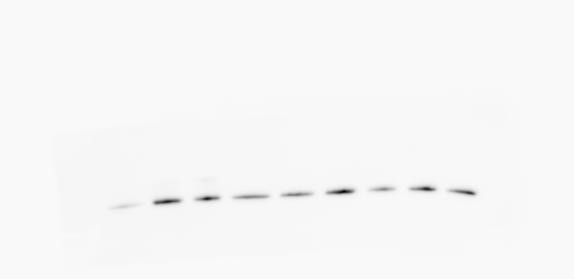


1 2 3

*21 kDa*


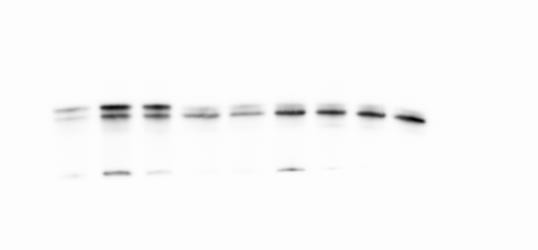


1 2 3

*21 kDa*


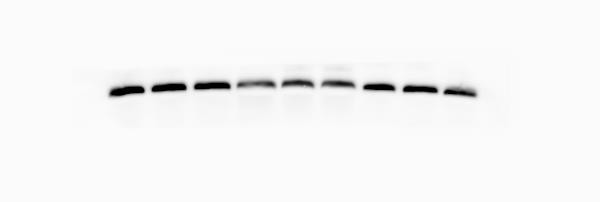


1 2 3

*50 kDa*

##### Cells RT4

RhoA


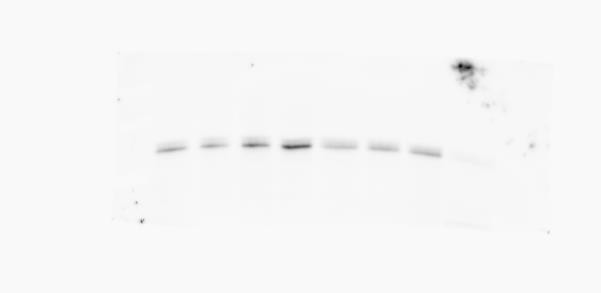


1 2 3

*21 kDa*

cortactin


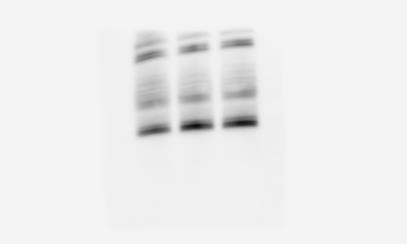


*85 kDa*

1 2 3


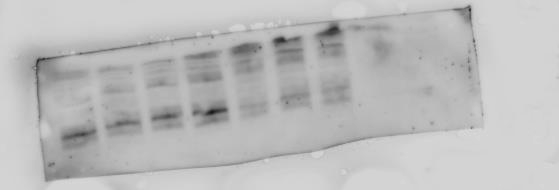

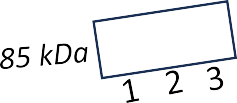


p-cortactin

Rac1/2/3

RhoC

Cdc42

Alpha-tubulin


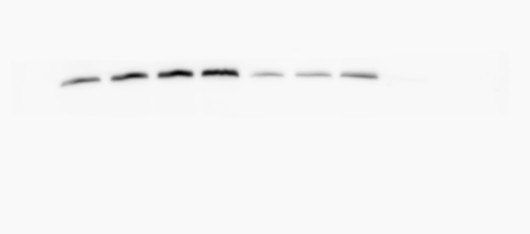


1 2 3

*50 kDa*


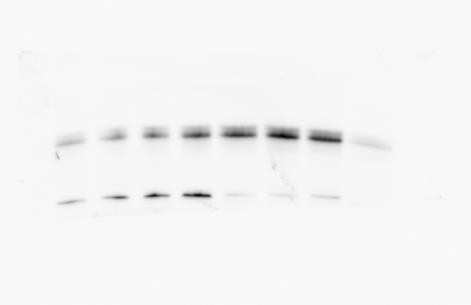


1 2 3

*21 kDa*


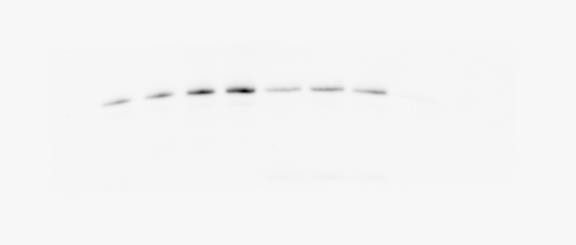


1 2 3

*21 kDa*


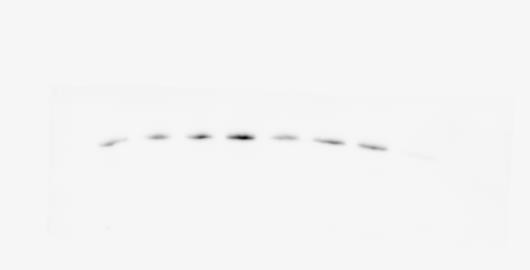


1 2 3

*21 kDa*

##### Cells NPU


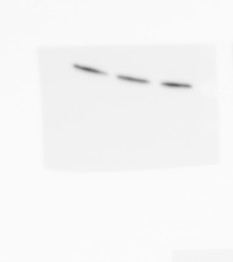

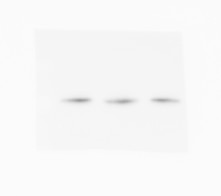


1 2 3

*21 kDa*

Rac1/2/3

*21 kDa*

RhoA

1 2 3


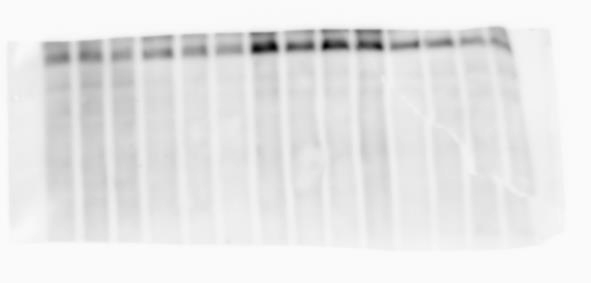


cortactin

*85 kDa*

1 2 3

p-cortactin


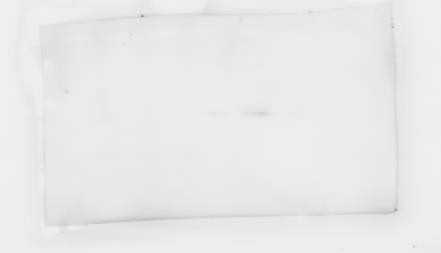


1 2 3

*85 kDa*

Cdc42


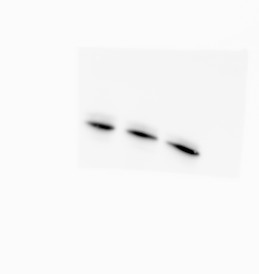


RhoC

1 2 3

*21 kDa*

Alpha-tubulin


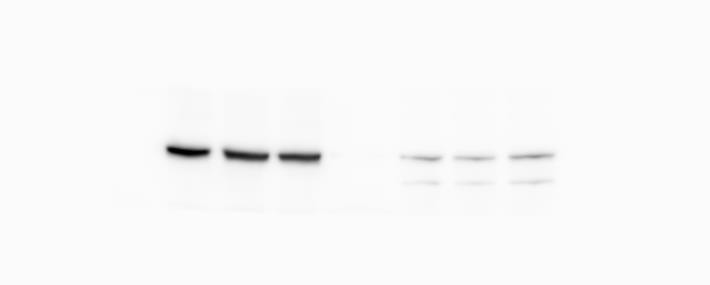


1 2 3

*50 kDa*


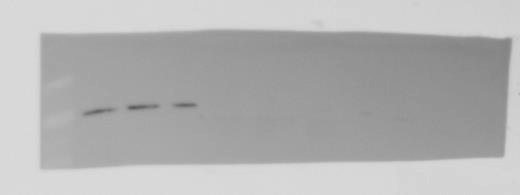


1 2 3

*21 kDa*


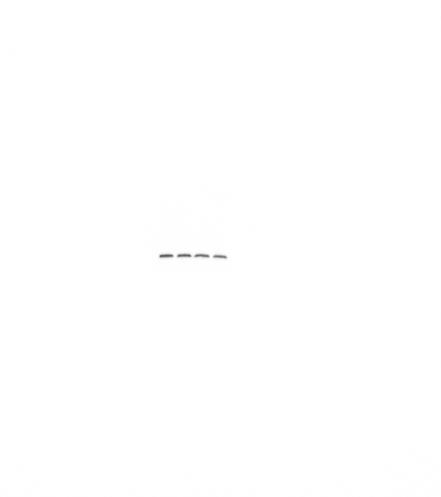


Alpha-tubulin

1 2 3 4

*50 kDa*

###### Legend:


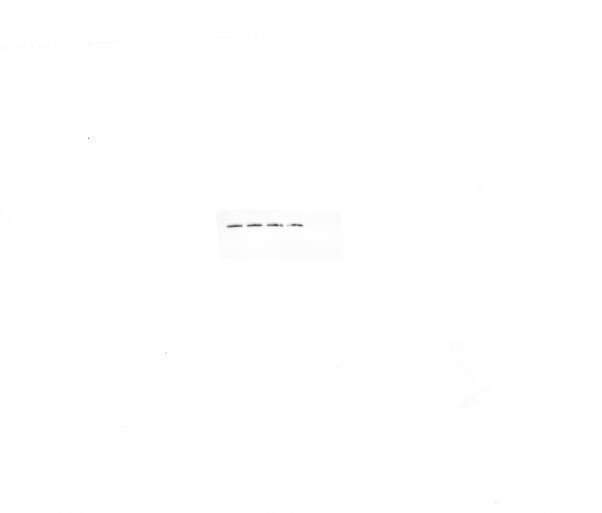

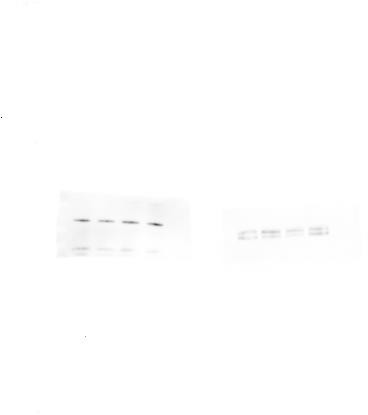


1 2 3 4

*21 kDa*

Rac123

*120 kDa*

1 2 3 4

FAK


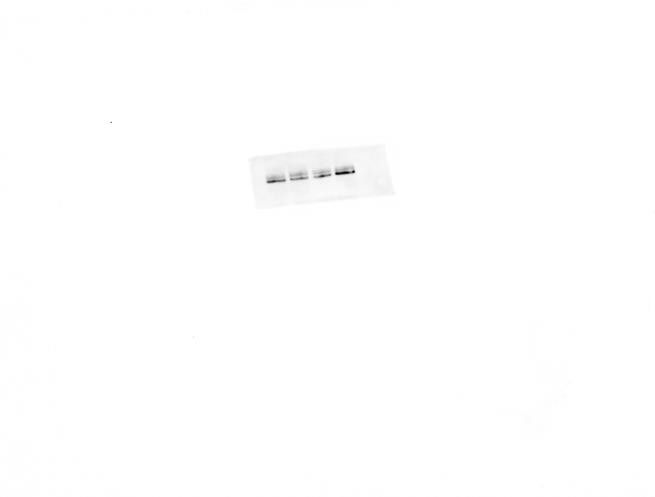

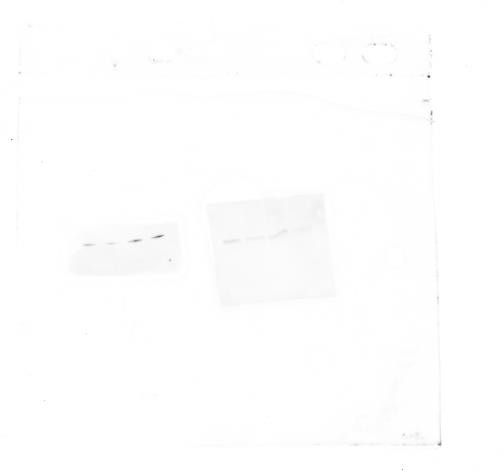


*21 kDa*

1 2 3 4

Cdc42

*50 kDa*

1 2 3 4

pFAK

**1 Defactinib + T24 2 PF573228 + T24**

**3 PND 1186 + T24**

**4 Control T24 cells**

MMP-2


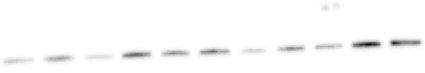


N-cadherin

1 2 3

*125 kDa*

Alpha-tubulin


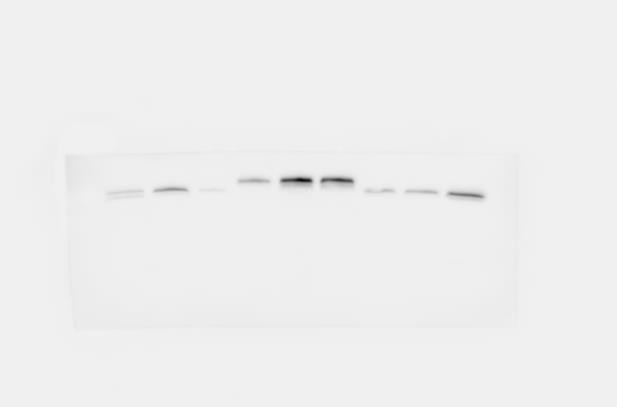


1 2 3

*72 kDa*

1 2 3

*50 kDa*


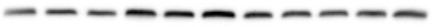


Alpha-tubulin

###### Legend:


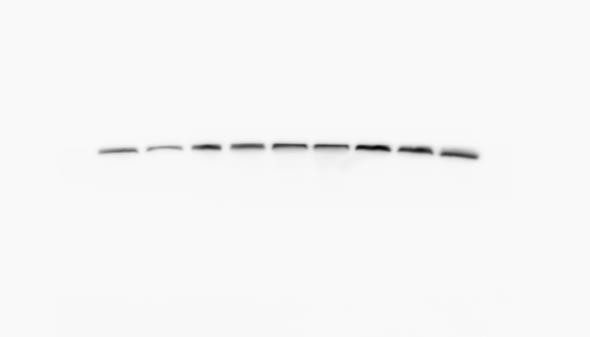


1 2 3

*50 kDa*

- - 1. **hAM homogenate**
    2. **hAM extract**
    3. **Control cells**

Figure 10E


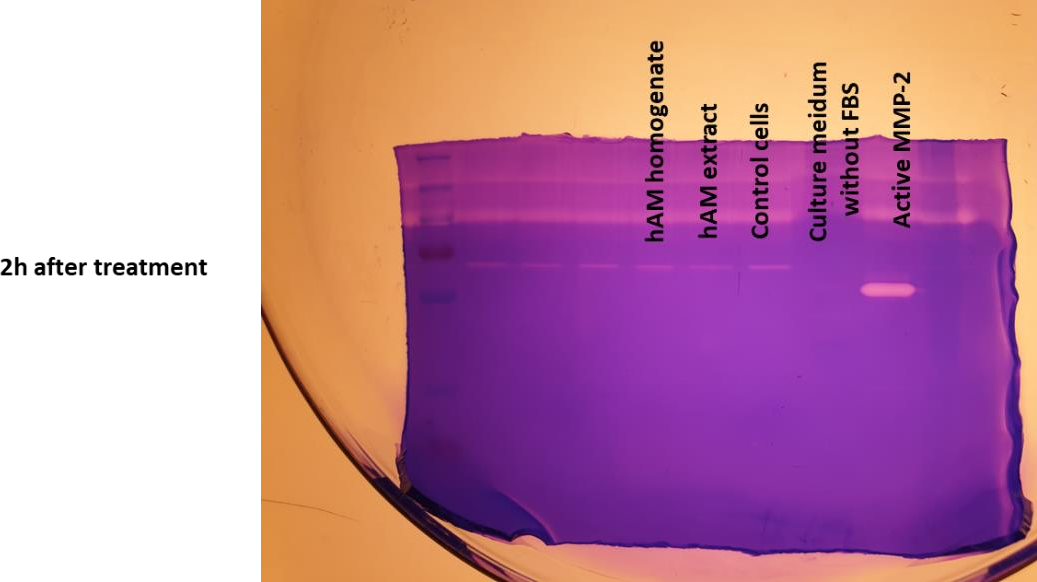

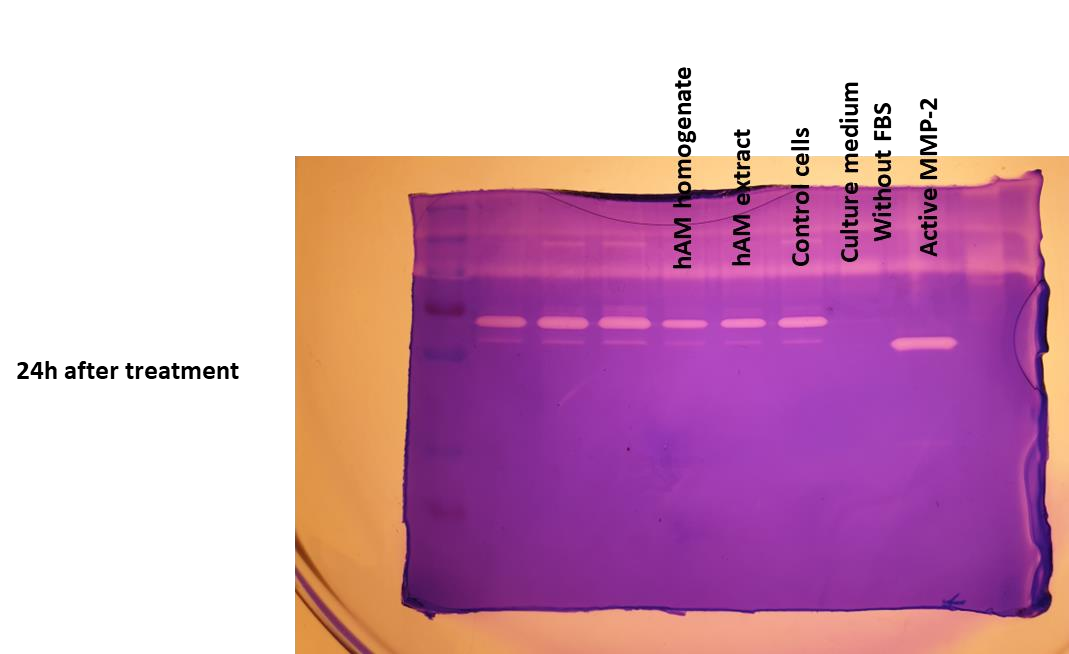


*250 kDa*

*130 kDa*

*100 kDa*

*70 kDa*

*55 kDa*

*35 kDa*

*25 kDa*

*250 kDa*

*130 kDa*

*100 kDa*

*70 kDa*

*55 kDa*

*35 kDa*

*25 kDa*

# Supplementary Figure 3. Images of visible membrane edges.

##### Cells T24

FAK

p-FAK

Alpha-tubulin


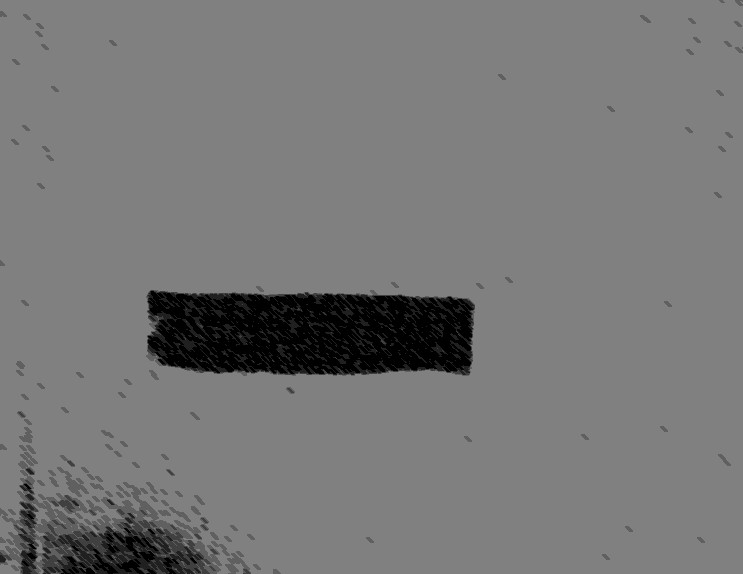


2 3


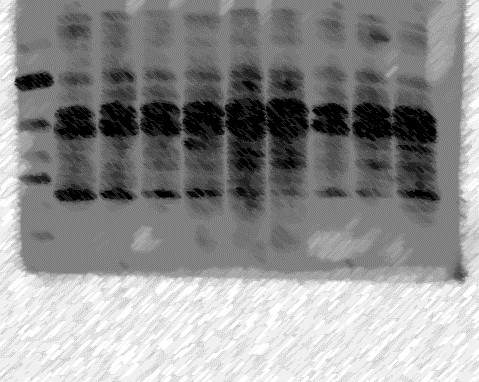


1 2 3


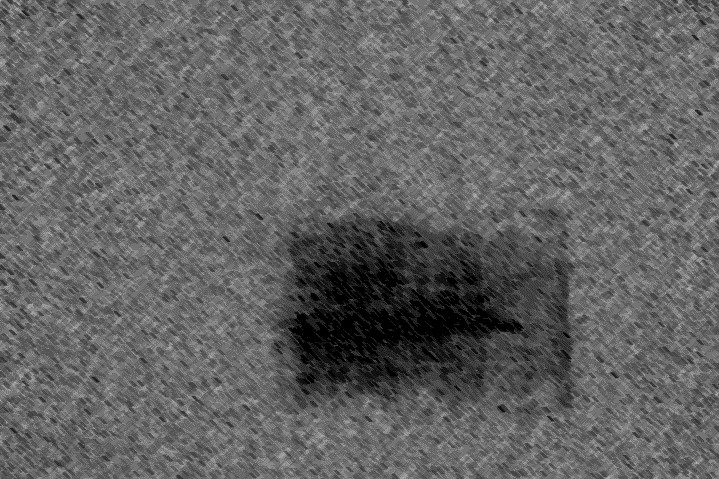


1 2 3

##### Cells RT4

FAK p-FAK Alpha-tubulin


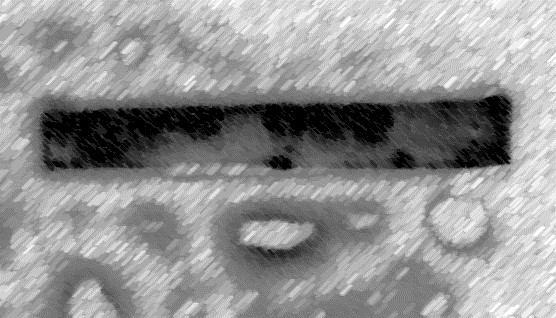


1 2 3


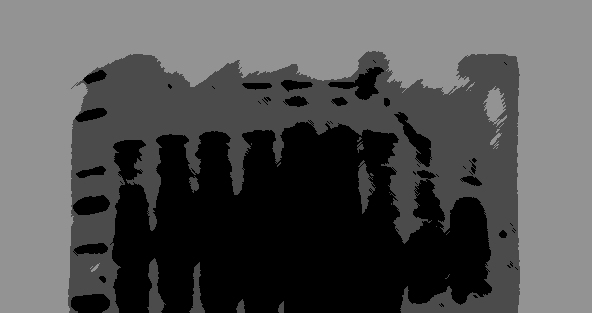


1


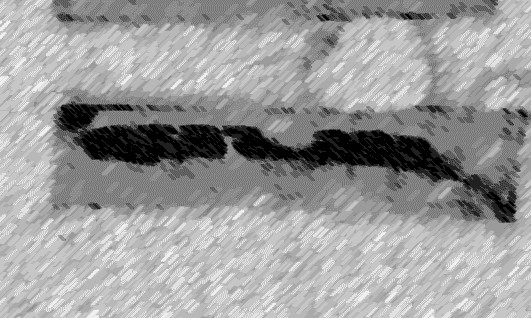


1 2 3

FAK

##### Cells NPU

p-FAK


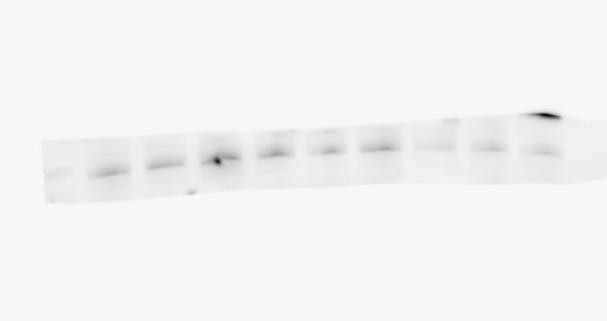


1 2 3

Alpha-tubulin


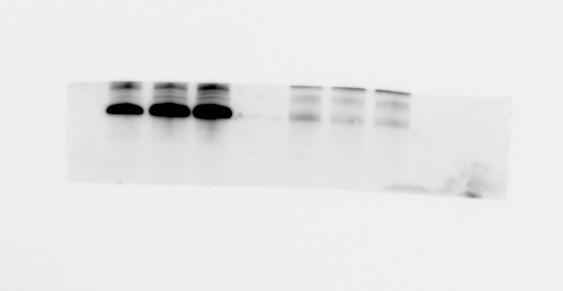


1 2 3


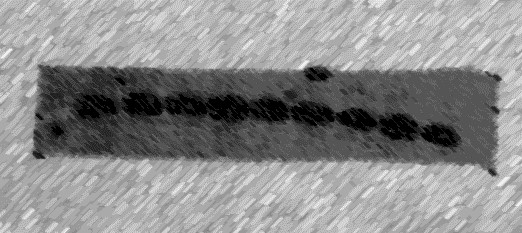


1 2 3

Pi3K p110a

##### Cells T24

Akt


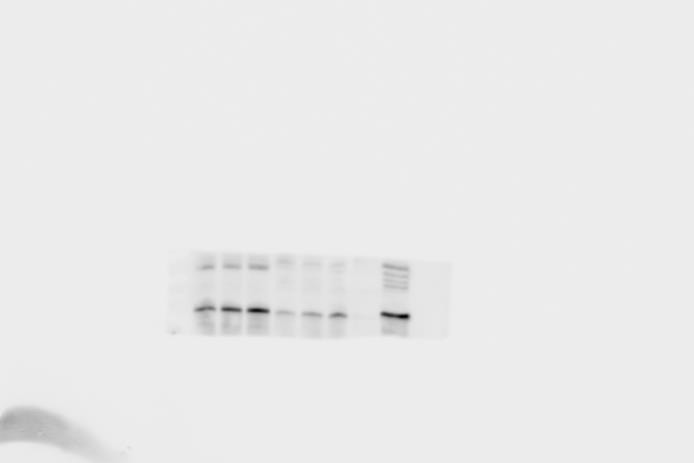


1 2 3

p-Akt


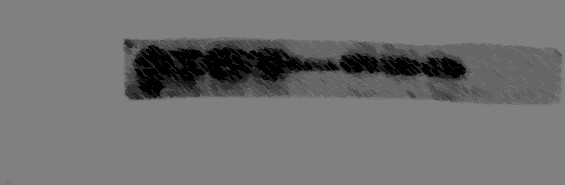


1 2 3

mTOR


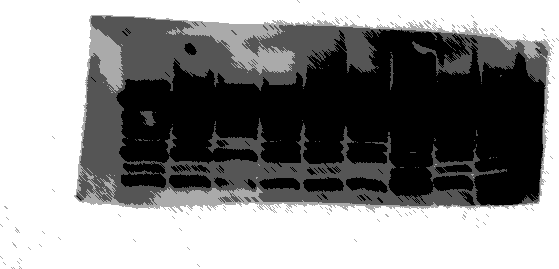

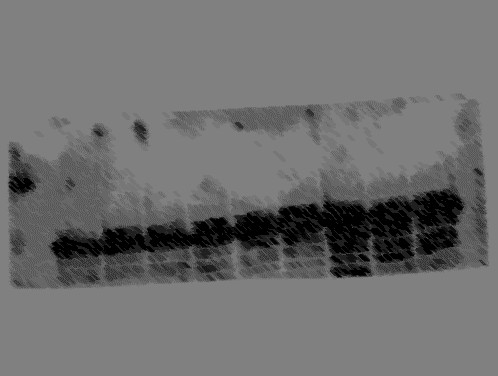

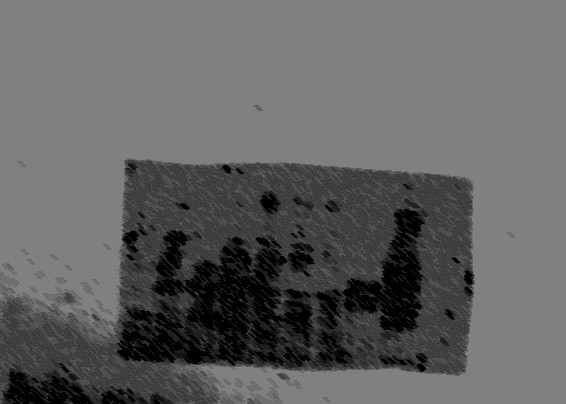


1 2 3


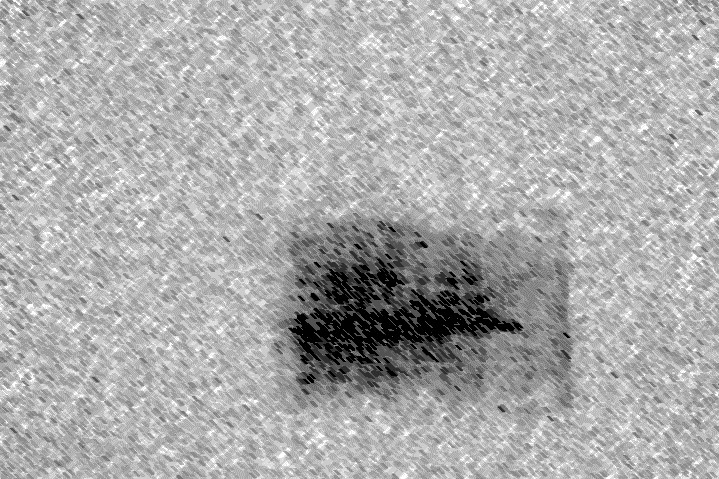


Alpha-tubulin

1 2 3

| 1 2 3 | p-mTOR |
| --- | --- |
|  | 1 2 3 |

Pi3K p110a

##### Cells RT4

Akt

p-Akt


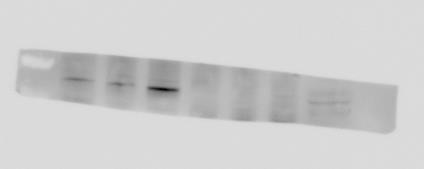


1 2 3


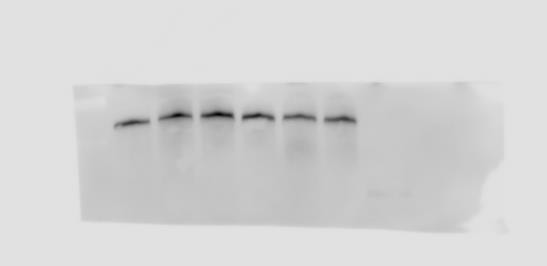


1 2 3


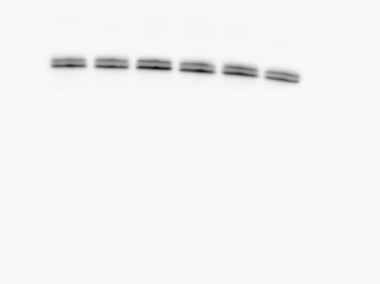


1 2 3

mTOR


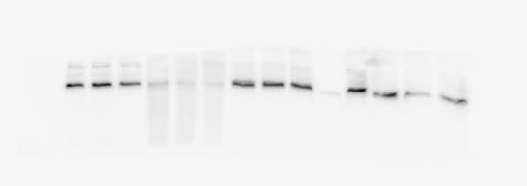


1 2 3

p-mTOR

Alpha-tubulin


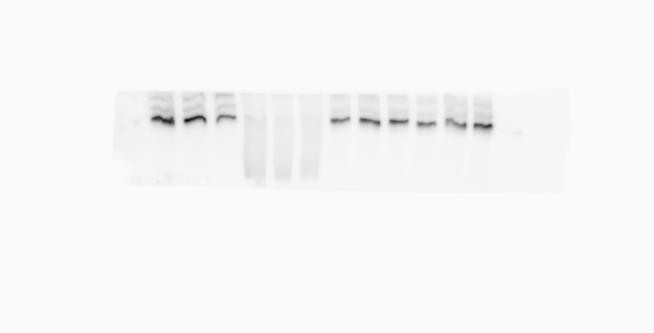


1 2 3


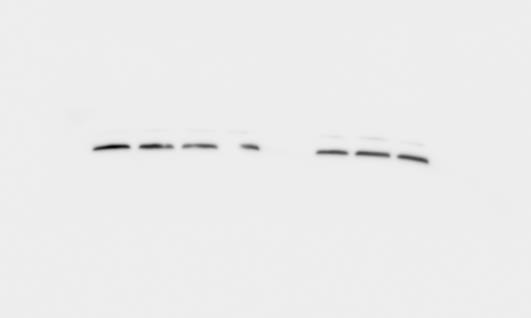


1 2 3

##### Cells NPU

Pi3K p110a


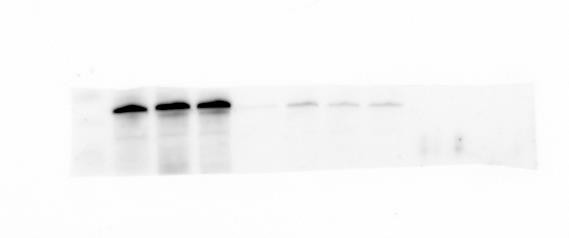


1 2 3

mTOR

Akt

p-mTOR


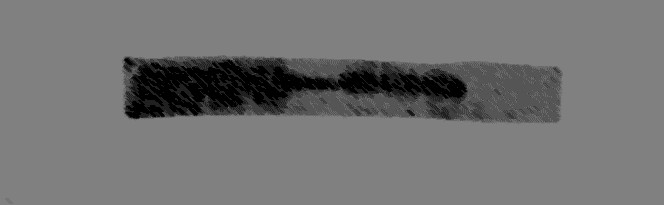


1 2 3

p-Akt


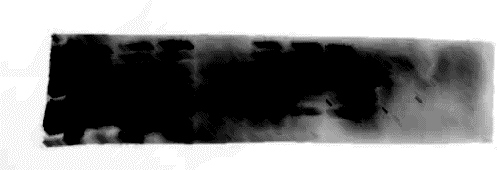


1 2 3

Alpha-tubulin


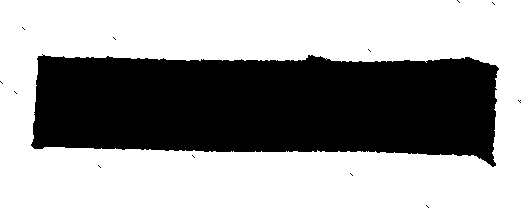


1 2 3


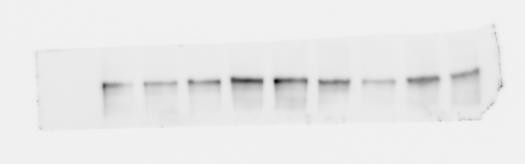


1 2 3


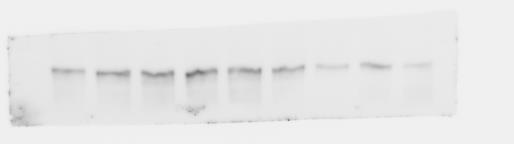


1 2 3

cortactin

##### Cells T24

p-cortactin

RhoA

Rac1/2/3


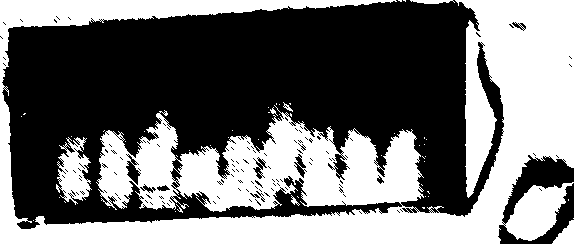

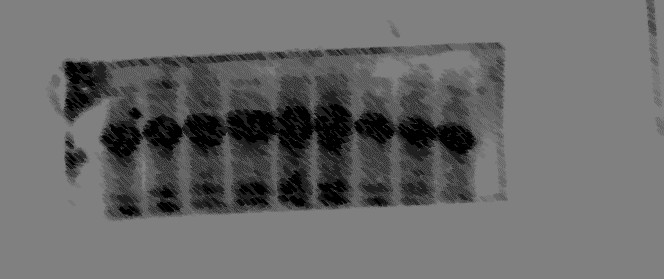


1 2 3


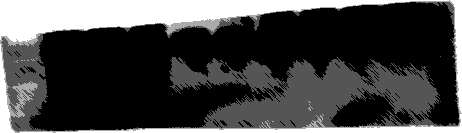


1 2 3


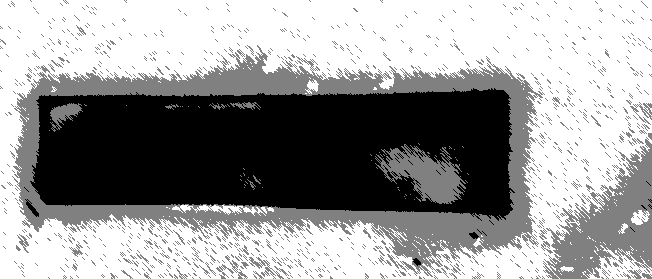


1 2 3

RhoC

Cdc42

Alpha-tubulin


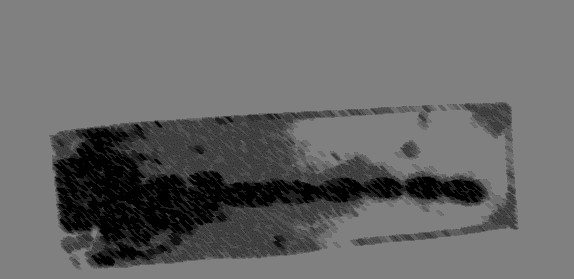


1 2 3


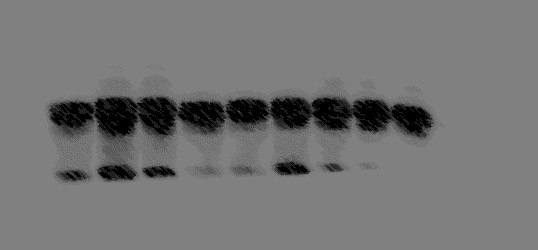


1 2 3


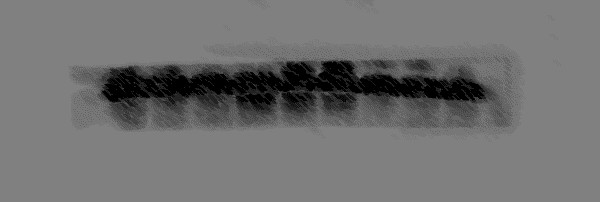


1 2 3

cortactin


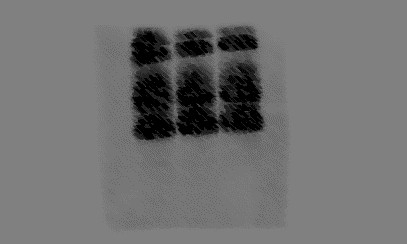


1 2 3

##### Cells RT4

p-cortactin


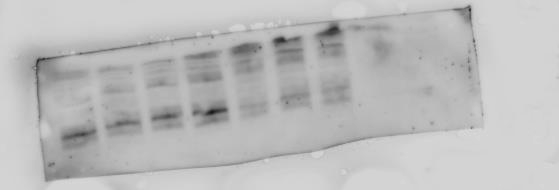

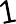

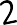

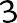


RhoA

Rac1/2/3

1 2 3

RhoC

Cdc42

Alpha-tubulin

1 2 3

1 2 3

1 2 3

1 2 3

cortactin

##### Cells NPU

- 1. ortactin

1 2 3

RhoA

1 2 3

Rac1/2/3

1 2 3

RhoC

1 2 3

Cdc42

Alpha-tubulin

1 2 3

1 2 3

Alpha-tubulin

1 2 3 4

###### Legend:

1 2 3 4

Rac123

1 2 3 4

FAK

1 2 3 4

Cdc42

1 2 3 4

pFAK

**1 Defactinib + T24 2 PF573228 + T24**

**3 PND 1186 + T24**

**4 Control T24 cells**

MMP-2

N-cadherin

1 2 3

1 2 3

Alpha-tubulin

Alpha-tubulin

1 2 3

###### Legend:

1 2 3

- - 1. **hAM homogenate**
    2. **hAM extract**
    3. **Control cells**

Figure 10E

# Supplementary Figure S4.

Multiple exposure images of high contrast membranes

FAK

##### Cells NPU

30 sec exposure

1 2 3

300 sec exposure

1 2 3

Contrast manually adjusted

1 2 3

Pi3K p110a Cells NPU

p-Akt

180 sec exposure

1 2 3

300 sec exposure

1 2

3

Automatic exposure by the machine (1min)

300 sec exposure

1 2 3

1 2 3

Contrast manually adjusted

1 2 3

1 2

3

600 sec exposure

##### Cells RT4

cortactin p-cortactin

1 2 3

30 sec exposure

75 sec exposure

1 2

3

Auto exposure (60 sec)

120 sec

360 sec

1 2

3

120 sec exposure

cortactin

##### Cells NPU

60 sec exposure

1 2 3

180 sec exposure

300 sec exposure

Supplementary figure 5. Full, unedited images of blots for all biological replicates.

FAK

Biological sample #B

1 2 3

*125 kDa*

FAK

Biological sample #C

*125 kDa*

1 2 3

FAK

Biological sample #D

*125 kDa*

2 3

FAK

Biological samples #E, #F, #G

*125 kDa*

1 1 1 3 3 3

p-FAK

Biological sample #B Biological sample #C

1 2 3

1 2 3

*50 kDa*

p-FAK

Biological samples #E, #F, #G

1 1 1

3

3 3

*50 kDa*

PI3K 110a

Biological sample #B

1 2 3

*110 kDa*

PI3K 110a

Biological sample #C

1 2 3

*110 kDa*

PI3K

Biological1s1am0aple #D

2 3

*110 kDa*

PI3K 110a

Biological samples #E and #F

1 1

3 3

*110 kDa*

*60*

*kDa*

Akt

Biological sample #C

1 2 3

Akt

Biological sample #D

2 3

*60*

*kDa*

Akt

Biological samples #E, #F, #G

1 1 1

3 3

3

*60*

*kDa*

Akt

Biological sample #H

1 2 3

*60*

*kDa*

P-Akt

Biological sample #B

1 2 3

*60*

*kDa*

P-Akt

Biological sample #C

1 2 3

*60*

*kDa*

P-Akt

Biological sample #D

2 3

*60*

*kDa*

P-Akt

Biological sample #H

1 2 3

*60*

*kDa*

mTOR

Biological sample #B Biological sample #C

1 2 3

1 2

3

*289 kDa*

mTOR

Biological samples #E, #F, #G

1 1 1 3 3 3

*289 kDa*

p-mTOR

Biological sample #B Biological sample #C

2 3

*289 kDa*

1 2 3

p-mTOR

Biological samples #E, #F, #G

1 1 1 3

*289 kDa*

Cortactin

Biological sample #B

1 2 3

*85 kDa*

Cortactin

Biological sample #D

2 3

*85 kDa*

Cortactin

Biological samples #E, #F, #G

1

1 1

3 3 3

*85 kDa*

p-cortactin

Biological sample #B

1 2 3

*85 kDa*

p-cortactin

Biological sample #D

2 3

*85 kDa*

p-

Biologicalcsoamrtpalcetsi#nE, #F, #G

1 1 1 3 3 3

*85 kDa*

RhoA

Biological sample #C

1 2 3

*21 kDa*

RhoA

Biological sample #D

2 3

*21 kDa*

RhoA

Biological samples #E, #F, #G

1

1 1 3

3 3

*21 kDa*

RhoC

Biological sample #B

1 2 3

*21 kDa*

RhoC

Biological sample #D

2 3

*21 kDa*

RhoC

Biological samples #E, #F, #G

1

1 1

3 3 3

*21 kDa*

Cdc42

Biological sample #C

1 2 3

*21 kDa*

Cdc42

Biological sample #D

2 3

*21 kDa*

CDC42

Biological samples #E, #F, #G

1

1 1

3 3 3

*21 kDa*

Rac123

Biological sample #C

1

2 3

*21 kDa*

Rac123

Biological sample #D

2 3

*21 kDa*

Rac12

Biological samp3les #E, #F, #G

1

1 1

3 3 3

*21 kDa*

N-cadherin

Biological samples #E, #F, #G and Biological sample #D

1 1

1 3 3 3

2 3

*125 kDa*

N-cadherin

Biological samples #B and #C

1 2 3 1 2 3

*125 kDa*

MMP-2

Biological samples #B

1 2 3

*72 kDa*

MMP-2

Biological samples #E and #F

1 1

3 3 1 2 3 2 3

*72 kDa*

Biological samples #H and #D

### Cells T24 (figures 6, 7, 8 and 10)

2 - extract hAM 3 - Control cells

tubulin

Biological sample #B

1 2 3

*50 kDa*

tubulin

Biological sample #C

1 2 3

*50 kDa*

tubulin

Biological sample #D

*50 kDa*

2 3

tubulin

Biological sample #H

1 2 3

*50 kDa*

tubulin

Biological samples #E, #F, #G

1 1 1 3 3 3

*50 kDa*

### Cells RT4 (Figure 6)

2 - extract hAM 3 - Control cells

FAK

Biological sample #B

1 2 3

*120 kDa*

FAK

Biological sample #C and #D

1 1 2 3

*120 kDa*

FAK

Biological sample #E

1 2 3

*120 kDa*

p-FAK

Biological sample #B

1 2 3

*50 kDa*

p-FAK

Biological sample #C and #D

1 1 2 3

*50 kDa*

p-FAK

Biological sample #E

1 2 3

*50 kDa*

PI3K 110a

Biological sample #B

1 2 3

*110 kDa*

PI3K 110a

Biological sample #C and #D

1 1 2 3

*110 kDa*

PI3K

Biologica1l1sa0maple #E

1 2 3

*110 kDa*

PI3K 110a

Biological sample #F

2 3

*110 kDa*

Akt

Biological sample #B

1 2 3

*60 kDa*

Akt

Biological sample #C and #D

*60 kDa*

1 1 2 3

Akt

Biological sample #E

1 2 3

*60 kDa*

p-Akt

Biological sample #B

1 2 3

*60 kDa*

p-Akt

Biological sample #E

1 2 3

*60 kDa*

mTOR

Biological sample #D

1 2 3

*289 kDa*

mTOR

Biological sample #E

*289 kDa*

1 2 3

mTOR

Biological sample #F

*289 kDa*

1 2 3

p-mTOR

Biological sample #B

1 2 3

*289 kDa*

1 2 3

*289 kDa*

p-

Biologicaml sTaOmRple #E

*289 kDa*

1 1 2 3

p-

Biological smamTpOleR#C and #D

cortactin

Biological sample #B

*85 kDa*

1 2 3

cortactin

Biological sample #F

*85 kDa*

1 2 3 1 2 3

Biological sample #E

cortactin

Biological sample #G

1 2 3

*85 kDa*

p-cortactin

Biological sample #B

*85 kDa*

1

2

3

p-cortactin

Biological sample #F

1 2 3

*85 kDa*

Biological sample #E

1 2 3

RhoA

Biological sample #F Biological sample #B

1 2 3 1 2 3

*21 kDa*

RhoA

Biological sample #E Biological sample #G

1 2

3 1 2 3

*21 kDa*

RhoC

Biological sample #F Biological sample #B

1

2 3 2 3

1

*21 kDa*

RhoC

Biological sample #E Biological sample #G

1 2

3 1 2 3

*21 kDa*

Cdc42

Biological sample #F Biological sample #B

1 3 1 2

2

3

*21 kDa*

Cdc42

Biological sample #E

1 2 3

*21 kDa*

Cdc42

Biological sample #G

1 2 3

*21 kDa*

Rac123

Biological sample #F Biological sample #B

1

2 3 2 3

1

*21 kDa*

Rac123

Biological sample #E Biological sample #G

1 2

3

1 2 3

*21 kDa*

### Cells RT4 (Figures 6, 7 and 8)

2 - extract hAM 3 - Control cells

tubulin

Biological sample #B

1 2 3

*50 kDa*

tubulin

Biological sample #E

*50 kDa*

1

2 3

tubulin

Biological sample #C and #D

1 1 2 3

*50 kDa*

tubulin

Biological sample #F

1 2 3

*50 kDa*

tubulin

Biological sample #G

*50 kDa*

| *120 kDa* | FAK  Biological sample #B and #C  1 2 3 1 2 3 | *50 kDa* | p-FAK  Biological sample #B and #D  1 2 3 1 2 | 3 |
| --- | --- | --- | --- | --- |

*110 kDa*

3

1 2

PI3K-110a

Biological sample #C

*110 kDa*

3

1 2

PI3K-110a

Biological sample #B

Akt

Biological sample #B and #C

1 2 3 1 2 3

*60 kDa*

p-AKT

Biological sample #B

1

*60 kDa*

2 3

mTOR

Biological sample #B and #D

1 2 3

1 2 3

*289 kDa*

p-mTOR

Biological sample #B and #D

1 2

3

1 2 3

*289 kDa*

p-AKT

Biological sample #C

1 2 3

*60 kDa*

### Cells NPU (Figure 8)

2 - extract hAM 3 - Control cells

Cortactin

Biological sample #B and #C

1

2 3

1 2

3

*85 kDa*

p-cortactin

Biological sample #B and #D

1 2

3

1

2

3

*85 kDa*

Cdc42

Biological sample #B

1 2

3

1 2

3

*21 kDa*

Rac123

Biological sample #C and #D

1 2 3

1 2 3

*21 kDa*

RhoC

Biological sample #B and #C

1 2

3

1 2 3

*21 kDa*

RhoA

Biological sample #B and #D

1 2 3

1 2 3

*21 kDa*

### Cells NPU (Figures 6, 7 and 8)

2 - extract hAM 3 - Control cells

tubulin

Biological sample #C

1 2 3

*50 kDa*

tubulin

Biological sample #B and #D

1 2 3

1 2 3

*50 kDa*

## Supplementary Figure 6. Original blots with molecular size markings from multiple independent experiments (#) to demonstrate the target antigen specificity.

#### FAK, Cell Signaling Technology #3285 – 125 kDa

FAK (#1)

FAK (#2)

FAK (#3)

1 2 3

250 kDa

130 kDa

100 kDa

1 1 2 3

130 kDa

250 kDa

1 2 3

1 2 3

100 kDa

130 kDa

#### p-FAK, Thermo Fisher Scientific #700255 – 50 kDa

p-FAK (#1)

p-FAK (#2)

p-FAK (#3)

1 2 3

1 2 3

1 2 3

55 kDa

#### PI3K 110alpha, Cell Signaling Technology #4249 – 110 kDa

PI3K 110a (#1)

PI3K 110a (#2)

PI3K 110a (#3)

1 2 3

1 2 3

100 kDa

130 kDa

1 2 3

130 kDa

250 kDa

Akt (#1)

Akt (#2)

Akt (#3)

1 2 3

25 kDa

35 kDa

55 kDa

70 kDa

100 kDa

130 kDa

250 kDa

1 2 3

55 kDa

70 kDa

1 2 3

55 kDa

70 kDa

100 kDa

130 kDa

250 kDa

P-AKT (#1)

P-AKT (#2)

P-AKT (#3)

1 2

3

1 2 3

10 kDa

15 kDa

25 kDa

35 kDa

55 kDa

70 kDa

35 kDa

1 2 3

55 kDa

1

2 3

70 kDa

mTOR (#1)

mTOR (#2)

mTOR (#3)

1

2

3

250 kDa

1 2

3

100 kDa

130 kDa

250 kDa

1

1 1

3

3

3

250 kDa

p-mTOR (#1)

p-mTOR (#2)

p-mTOR (#3)

1 1 2

3 1

2 3

250 kDa

1 2 3

1

2 3 1 2 3

250 kDa

1 2 3

250 kDa

Cortactin (#1)

Cortactin (#2)

Cortactin (#3)

1 2 3

1 2 3

55 kDa

70 kDa

100 kDa

1

2

3

55 kDa

70 kDa

100 kDa

130 kDa

250 kDa

1 2 3

55 kDa

70 kDa

100 kDa

130 kDa

250 kDa

p-cortactin (#1)

p-cortactin (#2)

p-cortactin (#3)

2 3

70 kDa

100 kDa

130 kDa

250 kDa

55 kDa

70 kDa

Different exp.

1 2 3

55 kDa

70 kDa

100 kDa

130 kDa

RhoA (#1)

RhoA (#2)

RhoA (#3)

1 2 3

10 kDa

15 kDa

25 kDa 35kDa

1 2 3

35 kDa

25 kDa

15 kDa

1 2 3 1 2 3

10 kDa

15 kDa

25 kDa

35 kDa

RhoC (#1)

RhoC (#2)

RhoC (#3)

1

2

3 1 2 3

10 kDa

15 kDa

25 kDa 35kDa

1 1 1 3 3 3

25 kDa

35 kDa

1 2 3 1

2 3

10 kDa

15 kDa

25 kDa

35 kDa

Cdc42 (#1)

Cdc42 (#2)

Cdc42 (#3)

10 kDa

15 kDa

2 3

25 kDa

35kDa

1 2

3

15 kDa

25 kDa

35 kDa

1 2 3

15 kDa

25 kDa

35 kDa

Rac123 (#1)

Rac123 (#2)

Rac123 (#3)

1 2 3

15 kDa

25 kDa 35kDa

1

2

3

1 2

3

15 kDa

25 kDa

35 kDa

1 2 3

15 kDa

25 kDa

35 kDa

N-cadherin (#1)

N-cadherin (#2)

N-cadherin (#3)

Technical replicate of biological samples

#E, #F, #G (Supplementary Figure 5)

1

1

1

3

3

3

100 kDa

130 kDa

250 kDa

Published paper by our research group (doi: 10.3389/fbioe.2020.554530)

55 kDa

70 kDa

100 kDa

130 kDa

250 kDa

This antibody was also used in a publication by our research group: https://doi.org/10.3390/ijms22115565

1 2 3 1 2 3

100 kDa

130 kDa

MMP2 (#1)

MMP2 (#2)

MMP2 (#3)

1

1

3 3

1 3

2

2 3

35 kDa

55 kDa

70 kDa

100 kDa

130 kDa

1

2

3

1

2 3

55 kDa

70 kDa

100 kDa

130 kDa

250 kDa

Different experiment, not included in study

100 kDa

70 kDa

55 kDa

35 kDa

Alpha tubulin (#1)

Alpha tubulin (#2)

Alpha-tubulin (#3)

1 2 3

1 2 3

55 kDa

70 kDa

35 kDa

1 2 3

55 kDa

and

1 2 3

55 kDa
